# Calcyphosine-like (CAPSL) is regulated in Multiple Symmetric Lipomatosis and is involved in Adipogenesis

**DOI:** 10.1038/s41598-019-44382-1

**Published:** 2019-06-11

**Authors:** Angie Lindner, Felix Marbach, Sebastian Tschernitz, Christine Ortner, Mark Berneburg, Oliver Felthaus, Lukas Prantl, Min Jeong Kye, Gunter Rappl, Janine Altmüller, Holger Thiele, Stephan Schreml, Julia Schreml

**Affiliations:** 1 Institute of Human Genetics, University Hospital of Cologne, Cologne, Germany; 2 Department of Dermatology, University Medical Center Regensburg, Regensburg, Germany; 3 Department of Plastic Surgery, University Medical Center Regensburg, Regensburg, Germany; 4 Center for Molecular Medicine Cologne (CMMC) and Department of Internal Medicine I, University of Cologne, Cologne, Germany; 5 Cologne Center for Genomics (CCG), University of Cologne, Cologne, Germany

**Keywords:** Autophagy, Mechanisms of disease, Gene expression, Mutation, DNA metabolism

## Abstract

Little is known on the causes and pathogenesis of the adipose tissue disorder (familial) Multiple Symmetric Lipomatosis (MSL). In a four-generation MSL-family, we performed whole exome sequencing (WES) in 3 affected individuals and 1 obligate carrier and identified Calcyphosine-like (*CAPSL*) as the most promising candidate gene for this family. Screening of 21 independent patients excluded *CAPSL* coding sequence variants as a common monogenic cause, but using immunohistochemistry we found that CAPSL was down-regulated in adipose tissue not only from the index patient but also in 10 independent sporadic MSL-patients. This suggests that CAPSL is regulated in sporadic MSL irrespective of the underlying genetic/multifactorial cause. Furthermore, we cultivated pre-adipocytes from MSL-patients and generated 3T3-L1-based *Capsl* knockout and overexpressing cell models showing altered autophagy, adipogenesis, lipogenesis and Sirtuin-1 (SIRT1) expression. *CAPSL* seems to be involved in adipocyte biology and perturbation of autophagy is a potential mechanism in the pathogenesis of MSL. Downregulation of CAPSL and upregulation of UCP1 were common features in MSL fat while the known MSL genes *MFN2* and *LIPE* did not show consistent alterations. CAPSL immunostainings could serve as first diagnostic tools in MSL clinical care with a potential to improve time to diagnosis and healthcare options.

## Introduction

Multiple Symmetric Lipomatosis (MSL, OMIM#151800) is a rare adipose tissue disorder of largely unknown etiology. Affected individuals show disproportionate, often disfiguring accumulation of subcutaneous adipose tissue. The MSL phenotype occasionally occurs together with neuropathic disorders. The genetic cause is heterogeneous and assumed modes of inheritance (MOI) include mitochondrial, autosomal recessive and likely also autosomal dominant inheritance. For patients with neurological affection, mutations in the mitochondrial genome (Myopathy with Ragged Red Fibres, MERRF, OMIM#545000) had initially been described, but could only explain few of the cases^1^. Recently, a homozygous mutation in one of the major genes causing Charcot-Marie-Tooth disease, Mitofusin 2 *(MFN2*), has been described^2^. Moreover, in a family with myopathy and lipodystrophy, a MSL like phenotypic appearance in non-dystrophic areas has been associated with a homozygous Lipase E (*LIPE*)-mutation^3^. The common pathogenetic mechanism leading to adipocyte dysfunction in these disorders is unknown. Here, we have identified Calcyphosine-like (*CAPSL*) as the single most interesting candidate gene in a four-generation family suffering from MSL. Although we could not find a second independent patient carrying a *CAPSL* mutation we have (i) found that CAPSL is not or scarcely expressed in familial as well as sporadic MSL (whereas it is normally expressed in control adipose tissue) and (ii) we found functional evidence for a connection between CAPSL, adipogenesis and MSL, respectively. Knowledge on CAPSL is scarce. A GEWAS linked the *CAPSL* locus to Type 1 diabetes^4,5^. CAPSL belongs to the calmodulin family and the calcium-binding EF-hand superfamily and it seems to interact with calmodulin^6^. For more information, see Supplementary Introduction.

## Results

### CAPSL was identified in a four-generation family affected by MSL

**Figure 1:**
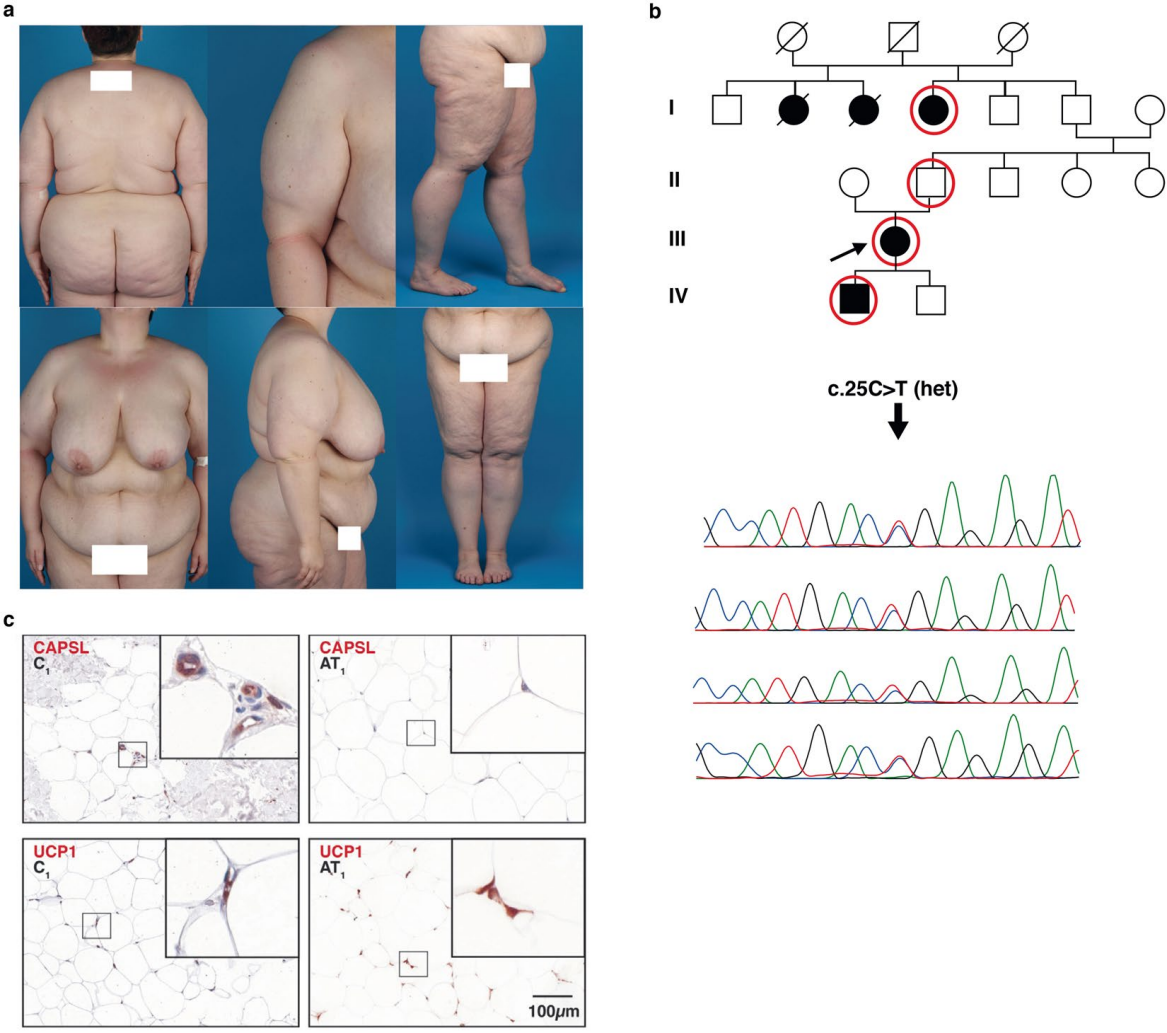
Phenotype, pedigree and immunohistology in a familial case of MSL. (**a**) Phenotype of the index patient in a MSL family. (**b**) Pedigree of the MSL affected family harboring a heterozygous rare missense variant in the *CAPSL* gene. Red circles denote family members who were examined using whole exome sequencing (WES). We validated variants of interest with Sanger sequencing. Sanger sequencing for the *CAPSL* variant c.25C > T in all 3 affected and one obligate carrier are shown. (**c**) Immunohistochemistry of CAPSL and UCP1 of control adipose tissue and of adipose tissue from a MSL affected region of the index patient. Upper row: CAPSL is absent in the index patient’s fat cells while it was found to be expressed in control fat. CAPSL was also not or rarely expressed in adipose tissue from sporadic MSL patients (Fig. 2). Lower row: UCP1 is strongly expressed in the MSL affected subcutaneous fatty tissue but not in control fatty tissue.

Genetic analysis in MSL is complicated by (1) the assumed autosomal-dominant mode of inheritance and both (2) the late age of onset and (3) the broad range of age of onset. For these reasons, we chose an “affected only” approach. In one family, we were able to perform WES on DNA extracted from whole blood samples in 3 affected individuals and one obligate carrier, which allowed for a very thorough reduction of variants (Fig. 1a,b, for WES analysis criteria, see Supplementary information and Fig. S1). None of them had a history of alcohol abuse. Among these, we identified *CAPSL* as the single most interesting candidate gene in the family. The observed rare variant (c.25C > T, p.R9*, NM_144647.3, Fig. 1b,) was validated using Sanger sequencing. It leads to a premature stop and haploinsufficiency of CAPSL in the affected patients. No additional germ line mutation in *CAPSL* could be found in the 21 unrelated available MSL patients from our cohort. None of the MSL affected or unaffected family members were reported to have signs of myopathy or neuropathy and we did not find evidence for heteroplasmy concerning the c.8344A > G MERRF genotype in the reads obtained by exome sequencing of blood DNA. Importantly, exome sequencing in the family did not reveal any mutations in the recently identified MSL genes *MFN2* or *LIPE*. The exome data did not suggest involvement of any other gene associated with lipodystrophy syndromes or overgrowth syndromes involving the mesenchymal lineages (e.g. phosphatase and tensin homolog *(PTEN*), Neurofibromin (*NF1*), Menin 1 (*MEN1*) and others, Fig. S1). We gathered human adipose derived stem cells (hASCs) from liposuction aspirate from the index patient and performed further functional analysis on these cells as described below.

### CAPSL is expressed in adipose tissue from controls but not in MSL patients

**Figure 2:**
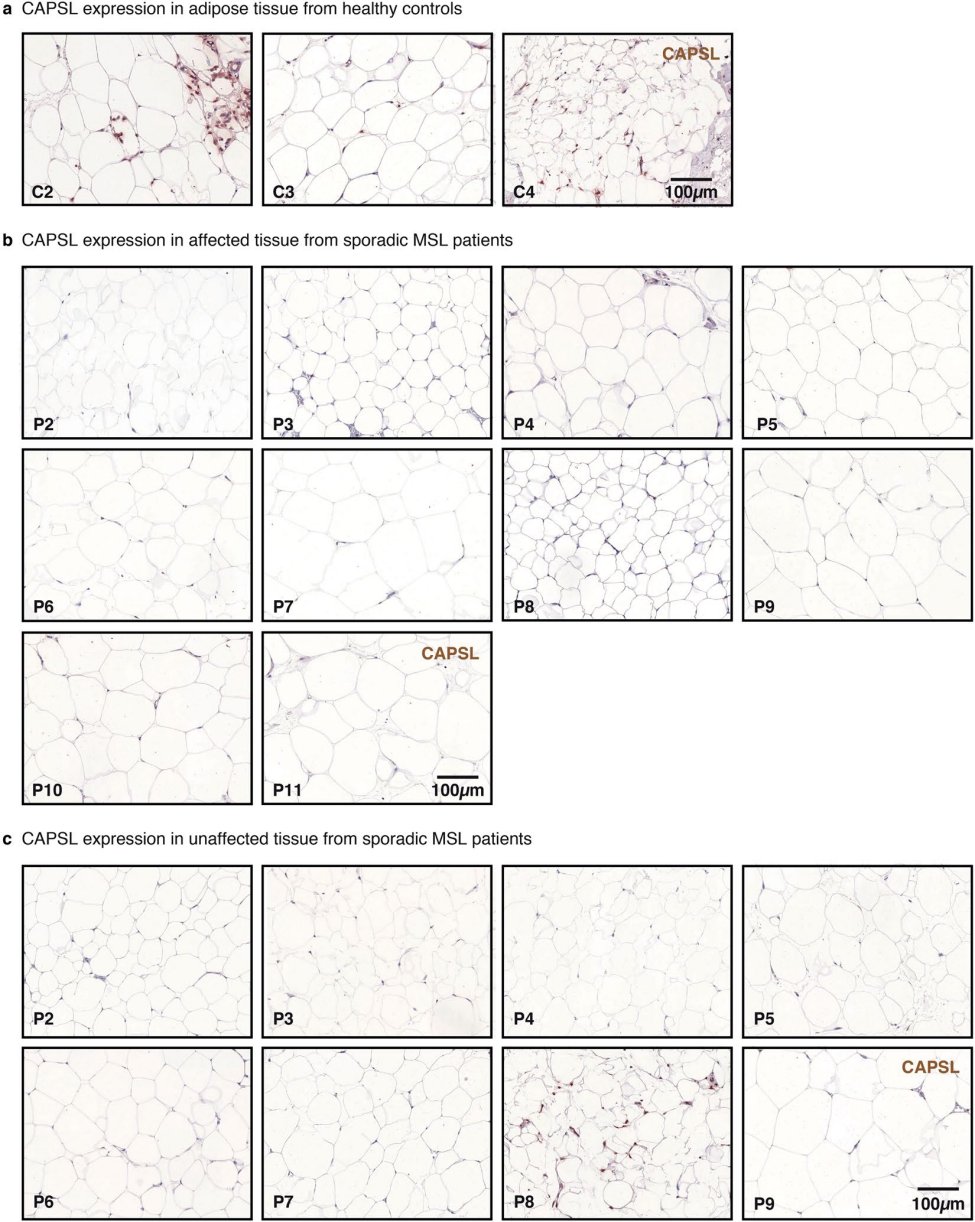
CAPSL expression in sporadic Multiple Symmetric Lipomatosis (MSL) tissue. Samples from healthy probands (**a**, n = 4) were examined and exemplary images are shown in the panel. Tissue from affected (**b**, AT, n = 11) and unaffected d (**c**, UT, n = 9) body regions were examined. Representative stainings from the index patient are presented in Fig. 1. CAPSL was regularly expressed in control adipose tissue (**a**), whereas little to no expression was observed in sporadic (**b**,**c**) and familial MSL (Fig. 1).

Immunostainings of native subcutaneous fat tissue showed expression of CAPSL in adipocytes of controls (n = 4, healthy probands), but hardly any expression in the affected fat tissue of the index patient (Fig. 1c, top). Staining for uncoupling protein 1 (UCP1) was positive in the index patient’s affected tissue but not in control adipose tissue (Fig. 1c, bottom). UCP1 is characteristic of brown adipose tissue and was previously reported by us and others to be expressed in affected adipose tissue from MSL patients^7,8^. Subsequent immunostaining of CAPSL (control adipose tissue in Fig. 2a) in adipose tissue from 10 sporadic MSL patients showed that CAPSL was not or scarcely expressed in all adipose tissue samples from affected regions (Fig. 2b). The same was true for samples from unaffected regions (Fig. 2c) of the same patients with the exception of patient 8, where CAPSL staining was positive in unaffected tissue, but not in the affected tissue. However, we did not detect a germline mutation in the coding sequence of *CAPSL*. To exclude somatic mutations of *CAPSL*, we examined the *CAPSL* sequence in affected regions of five MSL patients and could not detect any mutations. These findings suggest that CAPSL is – comparable to UCP1 – frequently altered in MSL. However, mutations of the coding sequence of *CAPSL* could only be found in the index family, suggesting other modes of regulation in the other examined patients.

We also examined LIPE and MFN2 expression in adipose tissue from MSL patients (Figs S2 and S3). LIPE was regularly expressed and there was no difference between controls and MSL patients (Fig. S2). MFN2 showed low or no expression in adipose tissue from controls and MSL patients (Fig. S3), respectively. However, one control showed relatively low expression and considerable natural variability exists due to ageing effects.

### *Capsl* regulates lipid accumulation in 3T3-L1-pre-adipocytes

**Figure 3:**
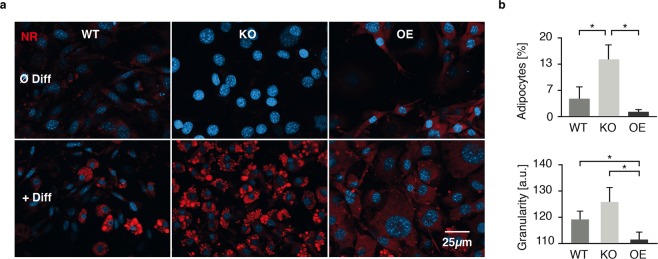
Involvement of CAPSL in adipogenesis. (**a**) 3 T3-L1 pre-adipocyte differentiation capability was analyzed in *Capsl* deficient (KO) and overexpressing (OE) cells via Nile Red (NR) staining and fluorescence microscopy. Increased NR positive lipid droplet formation was observed in KO cells compared with wild type (WT) cells, while OE cells showed an increased reddish background signal but formation of only few distinct lipid droplets in three independent experiments (n = 3). (**b**) To quantify the observed differences in adipocyte differentiation from fluorescence microscopy experiments, 3T3-L1 WT as well as OE and KO cells were analyzed using FACS. Size (FSC 0.9–2,5 × 10^5^) and granularity (preadipocytes: SSC 0.4–1 × 10^5^, adipocytes: 0.6–2.5 × 10^5^) were used to determine the fractions of 3T3-L1 populations and their lipid content within each sample. FACS measurements of the adipocyte population compared with total population (cells with increased granularity) for each cell line is shown in the upper graph. Adipocyte granularity is plotted in the lower graph. Experiments were repeated three times (mean ± SD) and analyzed by student’s t-test (*p < 0.05).

**Figure 4:**
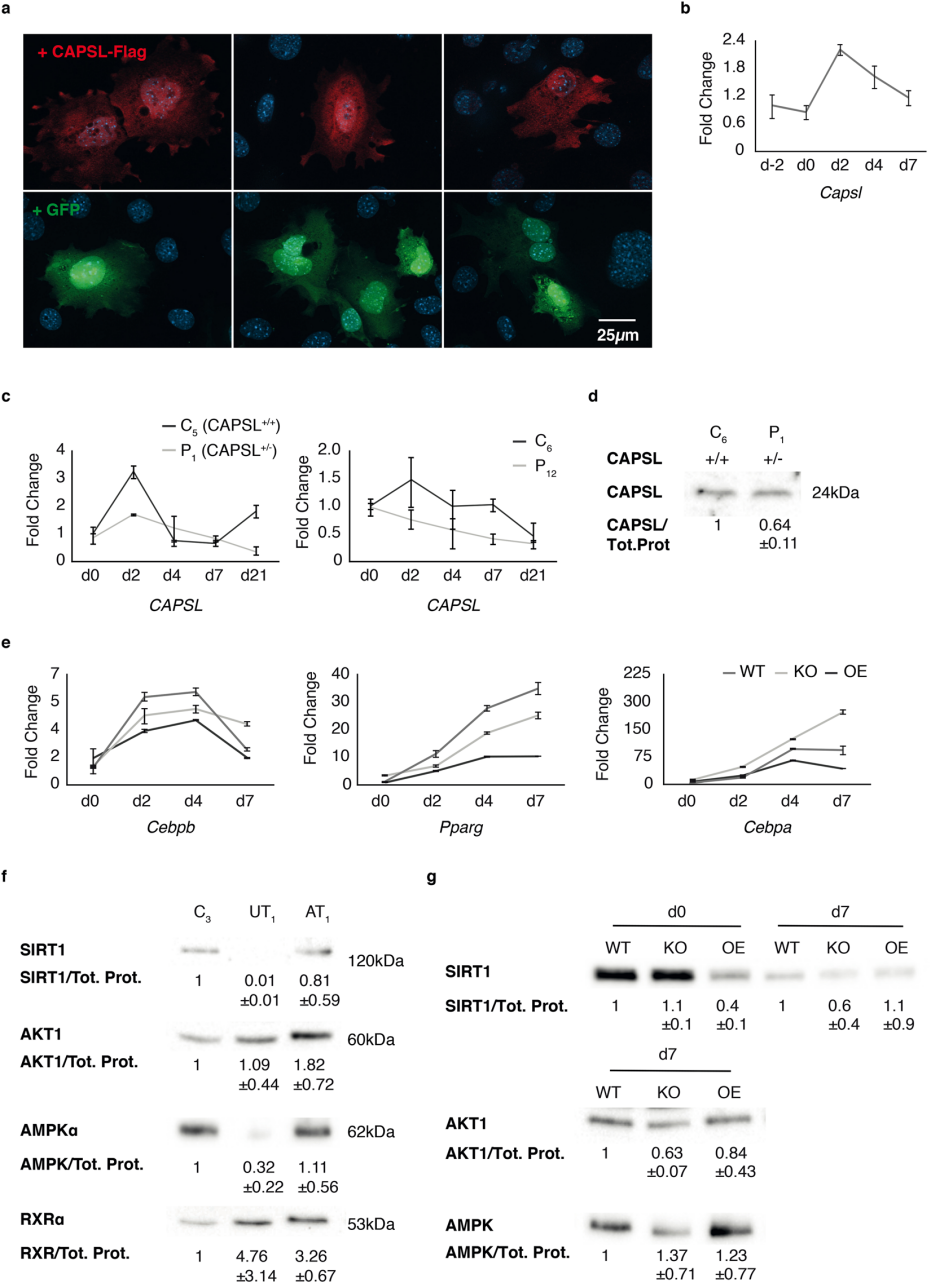
CAPSL expression and influence on genes involved in adipocyte differentiation and metabolism in 3T3-L1cells. (**a**) 3T3-L1 wild type (WT) cell were transiently transfected with a CAPSL-Flag construct. CAPSL protein distribution was visualized using α-Flag and α-rabbit secondary antibody (red, upper panel). The obtained signal was distributed diffusely in the cell without obvious subcellular predominance. Cells transiently transfected with a GFP expressing vector showed similar distribution (lower panel). (**b**) Murine WT pre-adipocytes were differentiated and RNA samples from different days were measured via qPCR. Three independent experiments were performed and representative graphs are shown. (**c**) *CAPSL* gene expression was measured via qPCR in hASCs of 2 healthy controls (C_2_ and C_3_), and in *CAPSL*^+/−^ (P_1_) adipocytes as well as in an independent MSL patient (P_2_). (**d**) CAPSL protein levels in *CAPSL*^+/+^ (control cells: C_3_) and *CAPSL*^+/−^ (index patient P_1_) adipocytes were examined using western blots (n = 2, mean ± SD). (**e**) 3T3-L1 WT, *Capsl* knockout (KO) and *Capsl* overexpressing (OE) cells were examined at day 0, 2, 4 and 7 of adipogenic differentiation. Histograms represent qPCR measurements of *Cebpb*, *Pparg* and *Cebpa*. (**f**) Expression of SIRT1, AKT1, AMPK and RXR was detected using western blot. Cells extracted from unaffected (UT) and affected (AT) MSL tissue from the index patient (*CAPSL*^+/−^) and control cells (C_3_) were examined. Representative bands are shown and quantification based on three independent experiments and total protein normalization was performed (mean ± SD). (**g**) 3T3-L1 undifferentiated preadipocytes (d0) and mature adipocytes (d7) were analyzed on western blots. The following protein amounts were examined in WT, KO and OE cells: SIRT1, AKT1 and AMPK. Quantification of intensity levels of three independent experiments and normalization relative to total protein (Tot. Prot) levels as well as SD values (mean ± SD) are shown below each representative protein band.

To further analyze a possible role of CAPSL in adipocyte biology and MSL in particular, we generated overexpressing and knockout cell lines from 3T3-L1-pre-adipocytes (see Methods and Fig. S4). Using Nile Red staining we investigated the ability of *Capsl* knockout (Capsl^KO^) and overexpressing (Capsl^OE^) 3T3-L1 cells (Fig. 3a,b) to differentiate into adipocytes. Capsl^KO^ cells showed enhanced propensity for differentiation, while Capsl^OE^ cells showed no proper lipid accumulation (Fig. 3a). Additionally, FACS analysis confirmed significantly increased lipid droplet accumulation in Capsl^KO^ cells (Fig. 3b). Capsl^OE^ cells, conversely, showed markedly reduced ability to differentiate (Fig. 3a,b) compared to Capsl^KO^ and wild type (WT) cells. Fluorescence microscopy on CAPSL-Flag construct expressing proliferating 3T3-L1 cells suggested that CAPSL was predominantly localized in the cytoplasm (Fig. 4a).

### CAPSL expression is upregulated at early stages of adipogenesis in 3T3-L1 cells as well as control hASCs but not in MSL hASCs

To analyze human *CAPSL* and murine *Capsl* expression during adipocyte differentiation we performed real time PCR (qRT-PCR) on d0, d2, d4 and 7 of differentiation in 3T3-L1 WT cells and d0, d2, d4 and d21 in control hASCs. We found that mRNA levels were low in WT proliferating 3T3-L1 cells, but peaked during the initial stages of differentiation (d2) (Fig. 4b). In human ASCs from healthy controls a similar temporal increase in *CAPSL* expression could be observed (Fig. 4c). Generally lower expression as well as a much lower peak during differentiation was observed in the index patients’ hASCs (Fig. 4c, left). These cells harbor a heterozygous *CAPSL* mutation leading to a premature translational stop at amino acid position 9 and to a reduction of CAPSL protein levels (Figs 1b and 4d). In line with the histological results, also hASCs from an additional independent MSL patient (without known *CAPSL* mutation), showed low *CAPSL* expression and no peak at d2 of differentiation (Fig. 4c, right).

### Capsl^KO^ cells display increased expression of the adipogenic factor *Cebpa* during differentiation

We performed qPCR on several markers of adipogenesis (*Cebpa, Cebpb, Pparg*) over the course of differentiation in 3T3-L1 cells. CCAAT/enhancer binding protein beta (*Cebpb*) was upregulated on d2 and d4 in all cell lines, but decreased towards the later stages of differentiation on d7. WT and Capsl^KO^ cells displayed a higher expression of *Pparg* between d0 and d7 as compared to overexpressing cells. For the late adipogenesis marker *Cebpa*, a marked increase in expression was observed in all cell lines between d0 and d4. *Cebpa* was also increased early in differentiation in Capsl^KO^ cells (Fig. S8b), and showed a sustained high expression into the late stages of adipocyte differentiation in this cell line, while stagnating or decreasing in WT and Capsl^OE^ cells (Figs 4e, S8a). The overexpression of *Capsl* in 3T3-L1 cells led to a generally decreased expression of the three differentiation markers.

### SIRT1 is differentially expressed in differentiated Capsl^KO^ cells and MSL hASCs

We examined expression of several proteins associated with fat cell metabolism and differentiation at baseline (undifferentiated) and at the end of the white adipocyte differentiation protocol, d7 and d21 respectively (Fig. 4f,g). In a previous study, we found that Sirtuin 1 (SIRT1) was among the strongest differentially expressed genes in MSL patient derived hASCs^9^. Accordingly, we found a downregulation in human *CAPSL* haploinsufficient adipocytes (Fig. 4f) as well as in 2 additional independent MSL hASC cultures (Fig. S5a). Moreover, the expression of SIRT1 was reduced in undifferentiated Capsl^OE^ cells and in mature Capsl^KO^ cells (Fig. 4g). For AKT serine/threonine kinase 1 (AKT1), we observed a trend towards lower expression in Capsl^KO^ cells in 3 independent experiments. AKT1 was upregulated in *CAPSL*^+/−^ patient hASCs as well as hASCs from two unrelated MSL patients (Figs 4f and S5a). For Protein kinase AMP-activated catalytic subunit alpha 1 (AMPK), no significant differences were observed between the cell lines (Fig. 4f). For AMPK results were inconsistent between the different patient hASCs (Figs 4f and S5a).

### CAPSL negatively regulates autophagy in 3T3-L1 cells

**Figure 5:**
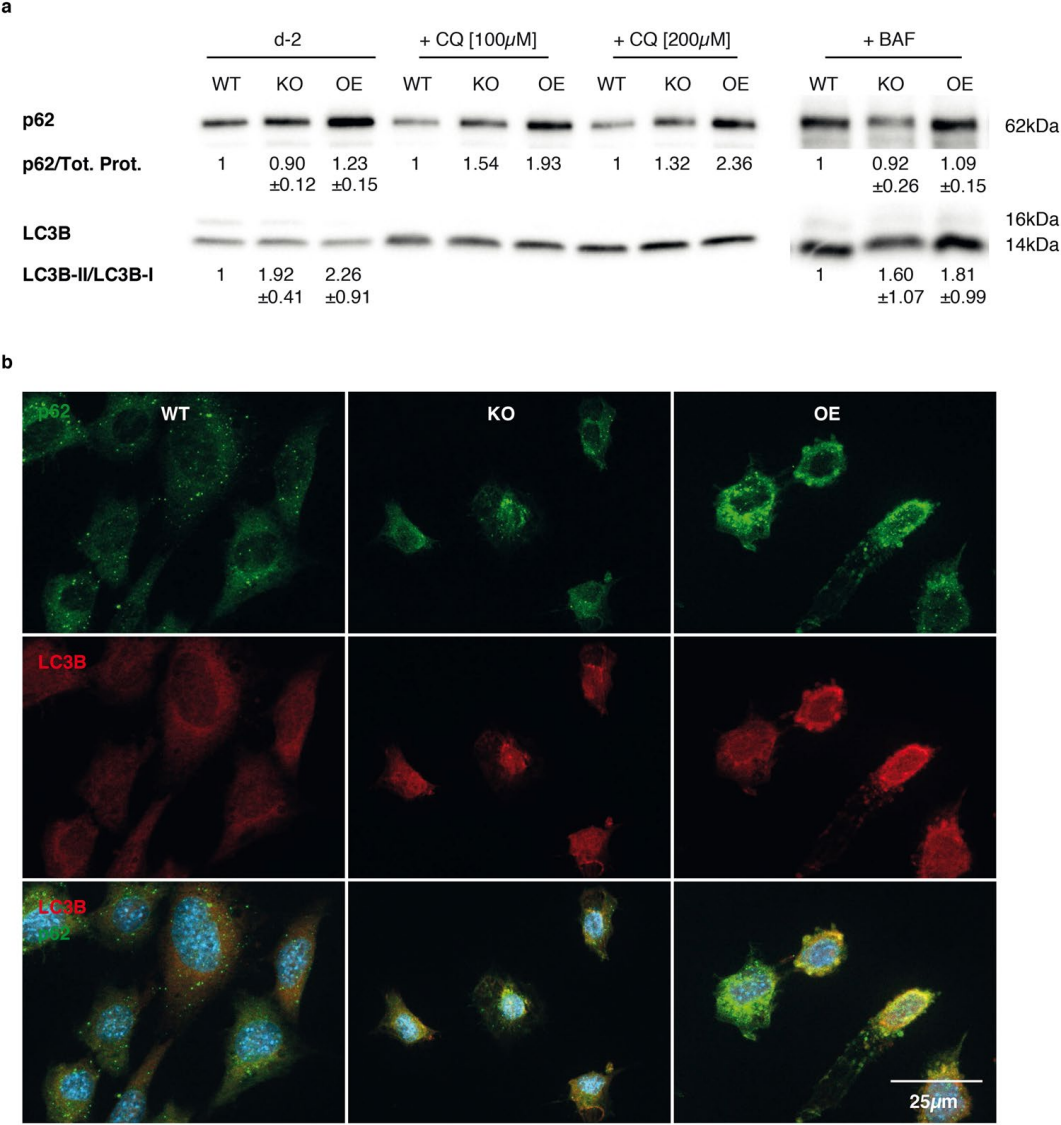
Autophagy flux in proliferating 3T3-L1 *Capsl* knockout (KO) and overexpressing (OE) pre-adipocytes. (**a**) Proliferating cells (D-2, n = 5, mean ± SD) were treated with 100 nM bafilomycin (BAF, n = 3, mean ± SD), 100 µM and 200 µM chloroquine (CQ, n = 1). Autophagosomal marker LC3B and autophagy substrate p62 were analyzed on western blots (**a**) and fluorescence microscopy (**b**).

**Figure 6:**
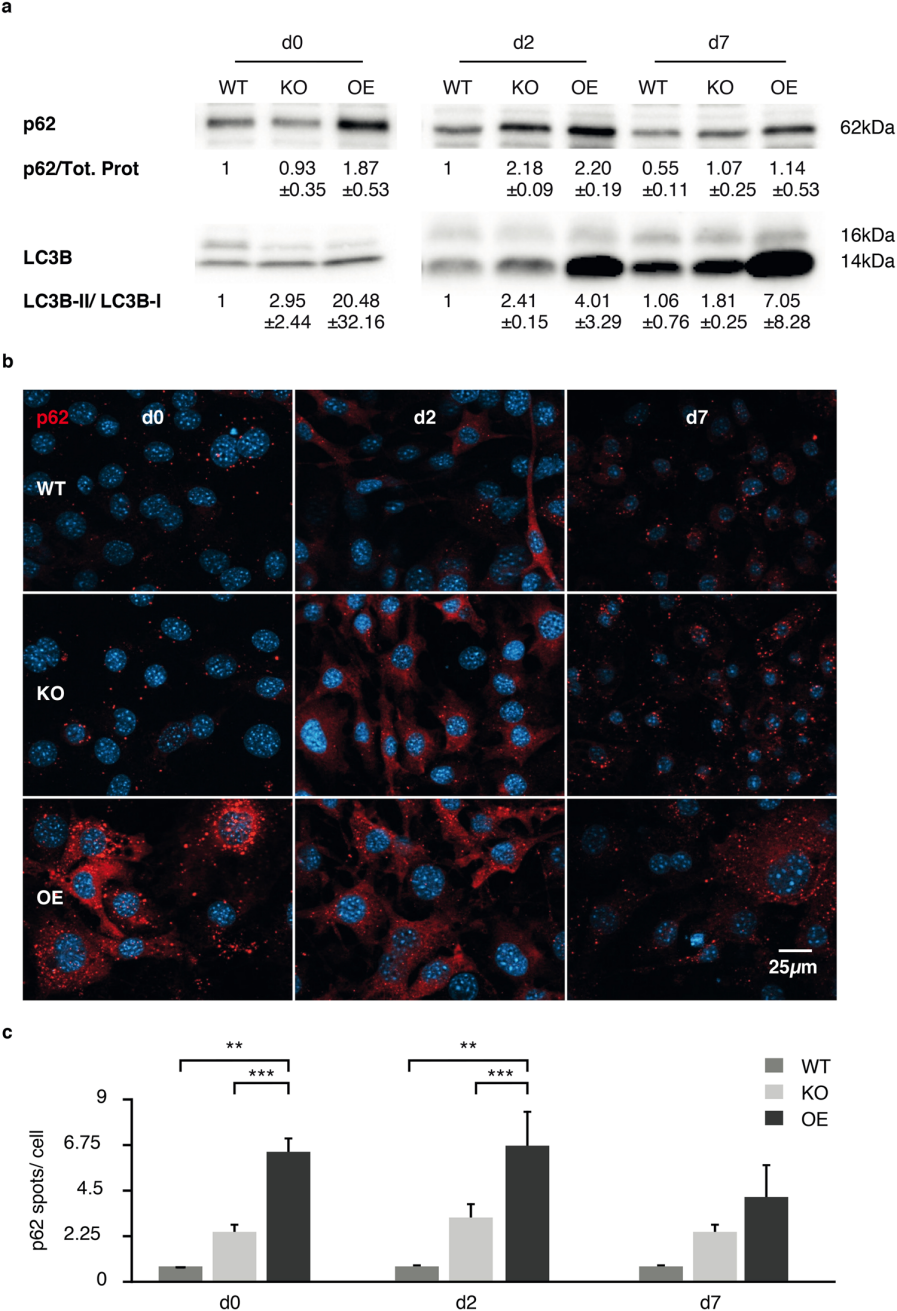
Autophagy flux during adipogenic differentiation. 3T3-L1 pre-adipocytes were differentiated *in vitro* and autophagy was analyzed via western blot (**a**) and fluorescence microscopy (**b**,**c** n = 3) at days 0, 2 and 7 of differentiation. Representative western blots are shown (D0 n = 3, D2-D7 n = 2) (**a**) and statistical significance was calculated by student’s t-test (**b,c**).

We analyzed the well-established marker for autophagosomes, Microtubule-associated protein 1 light chain 3 beta (LC3B) and the autophagy substrate Sequestosome 1 (p62) using immunoblot and fluorescence microscopy images of WT, Capsl^KO^ and Capsl^OE^ cells (Figs 5 and 6).

In untreated proliferating Capsl^KO^ and Capsl^OE^ cells, LC3B-II/LC3B-I levels were significantly increased compared to WT (Fig. 5a, WT and KO *p* < *0.01*; WT and OE *p* < *0.05*). Interestingly, Capsl^OE^ cells showed accumulation of p62 (*p* < *0.05*) and knockout cells showed lower p62 levels compared to WT (not significant) and OE (*p* < *0.01*) at baseline conditions (Fig. 5b). We could verify this result via fluorescence microscopy of p62 and LC3B (Fig. 5b). This indicated that autophagy flux may be reduced in Capsl^OE^ cells and possibly increased in Capsl^KO^ at baseline conditions.

To investigate autophagy flux at baseline conditions, 3T3-L1 pre-adipocytes were treated with chloroquine (CQ) and bafilomycin (BAF). These lysosomotropic reagents block a late step of autophagy. CQ prevents acidification and BAF impairs autophagosome-lysosomal fusion. The LC3B turnover assay in Fig. 5a shows autophagy flux in the analyzed cell lines after treatment^10^. We found increased LC3B levels in all cell lines after blockage of autophagy using BAF and CQ. Capsl^OE^ cells display the highest accumulation of p62 and LC3B, supporting the former results. In Capsl^KO^ cells, p62 was reduced compared to Capsl^OE^ cells, confirming autophagy is increased in the absence of CAPSL.

Furthermore, at the beginning of differentiation (Fig. 6a, d0) autophagy seems to be enhanced in Capsl^KO^ cells and blocked in Capsl^OE^ cells. During differentiation, autophagy seems to be blocked in both, Capsl^KO^ and Capsl^OE^ cells as shown by increased p62 and LC3B levels in immunoblot (Fig. 6a) and fluorescence microscopy images (Fig. 6b,c). In Capsl^KO^ cells, the autophagy block during differentiation was less pronounced compared to Capsl^OE^ cells.

Mechanistic target of rapamycin (mTOR) is a key player in autophagy regulation^11^. We have found evidence for dysregulation of AKT-mTOR-signaling on protein level in MSL patient cells (Figs 4f and S5a). Consequently, we examined mTOR-signaling via the mTOR Complex 1 (mTORC1) in our CAPSL 3T3-L1 cell lines by measuring phosphorylation of Ribosomal protein S6 kinase B1 (p70S6K) phosphorylation using immunoblot. However, mTOR activation was low in all examined cell lines (Fig. S5b), complicating proper interpretation and precluding further in-depth experiments.

### *Capsl* deficient cells are more resistant to oxidative stress but not ER stress

To test how *Capsl* deficient and overexpressing cells cope with ER and oxidative stress, we used MTT assays after treatment with ER stress inducers thapsigargin and tunicamycin as well as oxidative stress inducer H_2_O_2_ (Fig. S6a). Compared to WT, Capsl^KO^ cells were more resistant towards oxidative stress induced by H_2_O_2_ in 3 independent experiments. There was no difference in response towards ER-stress between *Capsl* deficient and WT cells in the MTT assays and qPCR (Fig. S7). Additional immunoblot analysis showed no difference in PDI expression levels between the cell lines (Fig. S6b) but increased levels of Heat shock protein 5 (BIP) and Endoplasmic reticulum to nucleus signalling 1 (IRE1a) were observed in Capsl^OE^ and Capsl^KO^ pre-adipocytes (Fig. S6b).

## Discussion

MSL occurs isolated or as an additional phenotypic feature in rare cases of neurological, myopathic or lipodystrophic syndromes. The underlying genetic factors are heterogeneous and different modes of inheritance apply. Mutations in *MFN2, LIPE* and *LMNA*^12–14^ lead to energy imbalance and accumulation of dysfunctional mitochondria. Enhancement of ageing related changes in different organelles have been demonstrated. The question remains what might be the common pathogenic mechanism leading to regional adipose tissue overgrowth. Identifying the genetic cause for “MSL-only” (without neuropathic or myopathic phenotype) patients might therefore provide new insights into the specific alterations affecting the adipocyte in the course of these diseases.

Using WES on venous blood DNA in 3 affected and an obligate carrier in a four-generation MSL family, we identified *CAPSL* as the most promising candidate gene in this family. CAPSL was hardly or not at all expressed in histological samples of subcutaneous adipose tissue in 10 patient samples of sporadic MSL cases. These results strongly suggest that CAPSL is regulated in sporadic MSL cases. However, we could not find germline mutations in *CAPSL* in 21 independent MSL patients, including 8 of the 10 patients from the histological study. Furthermore, we could exclude somatic mutations in hASCs harvested from affected regions of 5 patients. We cannot prove beyond any doubt that *CAPSL* germline mutations are a monogenic cause of MSL or are causally related to the MSL phenotype in the index family. However, the interesting immunohistochemistry prompted further functional studies discussed below and suggests a link between CAPSL deficiency and MSL. Possible mechanisms to consider are post-transcriptional regulation in the adipose tissue of MSL patients or alternative regulation at the transcriptional level. On a genetic level, mutations outside of the sequenced region of the gene or variants affecting transcriptional suppressors are conceivable. Future studies are required to investigate regulatory mutations affecting CAPSL expression in MSL cases.

We further analyzed the interesting histological findings of reduced CAPSL expression and used 3T3-L1 pre-adipocyte cell models to compare the effect of both, *Capsl* deficiency and overexpression. Capsl^KO^ adipocytes displayed a higher differentiation efficiency compared to the WT cells and overexpressing cells showed decreased adipogenesis. We found only low expression levels of murine *Capsl* and human *CAPSL* mRNA in 3T3-L1 cells and hASCs before initiation of differentiation. Expression was, however, reproducibly induced around d2 of differentiation in both, the murine and the human cell model and dropped back to pre-treatment levels at the end of differentiation. This suggested that CAPSL might be especially important in the very early stages of adipocyte differentiation. This idea was supported by analysis of the adipogenesis markers *Cebpb*, *Cebpa* and *Pparg* over the course of differentiation. *Cebpb*, which is induced in wild type at early stages of adipogenesis and lower expressed at late stages^15^, was higher expressed in mature knockout adipocytes. It has recently been reported that expression of *Pparg* was elevated upon the increase of intracellular Ca^2+^ levels in 3T3-L1-cells^16^ and that this might be one mechanism by which Ca^2+^ led to enhanced adipogenesis. *Pparg* was lower expressed in our *Capsl*^*KO*^ compared to WT cells. *Pparg* is induced by *Cebpb*^15^ and needed to sustain a differentiated cell state. Both, *Cebpa* and *Pparg* have been shown to be sufficient to initiate transdifferentiation of pluripotent stem cells into adipocyte like cells^17,18^. We found that in Capsl^KO^ cells *Cebpa* levels were higher compared to WT and increased during differentiation supporting a higher differentiation signal in knockout cells. *Capsl*^*OE*^ cells, on the other hand, expressed continuously lower levels of all examined markers which is in accordance with their impaired differentiation ability. In summary, these results suggest an inhibitory role of CAPSL in adipocyte differentiation.

The process of differentiation requires complex changes in expression patterns and a refined spatial and temporal interplay of many cellular organelles and factors. We performed experiments covering several essential cellular mechanisms known to influence adipocyte differentiation and/or mechanisms that have been suspected to be involved in MSL pathogenesis with a focus on energy and adipocyte metabolism signaling, autophagy and cell stress. Among several core proteins involved in lipid metabolism, SIRT1 showed the most interesting results. It was differentially expressed in murine *Capsl* and human *CAPSL* mutated cells as well as in independent MSL patient cells. For the other analyzed proteins, results varied between cell lines or between human and murine models. More patient hASCs need to be examined in future experiments to delineate natural variability in signaling cascades from true disease effects. SIRT1 is a nuclear protein involved in the regulation of many cellular processes including ageing/longevity and glucose homeostasis. Importantly it has been shown to inhibit PPARG-signaling and to increase white adipocyte lipid storage mobilization^19^. SIRT1 is also an important activator of the AMPK-complex. The AMPK-complex functions as a kinase relating cellular and environmental stress to signaling cascades aimed at counteracting e.g hypoxia or heat shock^20^. It is involved in fatty acid metabolism, autophagy and cell growth regulation through Tuberous sclerosis 2 (TSC2)/mTOR signaling^21^. Both mechanisms, autophagy and mTOR signaling have been previously implicated in the pathogenesis of adipocyte disorders. In specific, mTOR inhibition by SIRT1-AMPK signaling has been shown to be involved in an adaptive process during cell stress to ensure cell survival by regulating energy expenditure, autophagy and other cellular processes^22,23^. We found a trend towards increased p70S6K phosphorylation in Capsl^OE^ cells, which were proliferating or harvested at d0 of differentiation, compared with Capsl^KO^ and WT cells but results were not significant. However, baseline activation levels were low and effective rapamycin treatment was hard to establish due to toxicity. Further experiments (e.g. using SIRT1 activation and p70S6K readout) will be needed to find out whether mTOR signaling is involved in any relevant manner in the differentiation phenotype of the cells.

Examining autophagy flux at baseline and during differentiation, we found that in proliferating cells and cells at d0 of differentiation autophagy flux was increased in Capsl^KO^ cells and decreased in Capsl^OE^ cells. It is well known, that autophagy is upregulated at early phases of adipogenesis and subsequently downregulated. Analysis of autophagy during differentiation again reflected a defective autophagy flux. It can be assumed that autophagy is not adequately downregulated during adipogenesis in Capsl^KO^ cells. Thus, our results not only show an inhibitory role of CAPSL in adipogenesis, but also in autophagy. Since autophagy is needed for adipogenesis, we speculate that CAPSL might influence adipogenesis negatively via autophagy flux regulation. Future experiments are needed to elucidate the exact role of CAPSL in this cellular process.

We observed a conversion from high basal SIRT1 expression and increased autophagy flux towards decreased SIRT1 expression and decreased autophagy after differentiation in Capsl^KO^ cells. Proliferating Capsl^OE^ cells exhibit an opposite pattern in SIRT1 expression and autophagy activity. Reduced SIRT1 expression was reported to decrease H_2_O_2_-induced autophagy in human and mouse embryonic stem cells^23^ and autophagy regulation during oxidative stress was demonstrated to be modulated by calcium levels in human endometrium derived stem cells^24^. We found that low SIRT1 expressing Capsl^KO^ cells were indeed able to cope better with the H_2_O_2_ treatment.

Our study provides new insights into the genetics of familial and sporadic MSL and possible functions of CAPSL in mouse and men. The genetic causes of MSL without neurological phenotype are yet to be elucidated. Our results suggest that *CAPSL* coding sequence mutations are not a frequent cause of MSL. However, CAPSL seems to be regulated frequently in MSL tissue, prompting the question of possible recurring regulatory mutations in sporadic MSL cases which exceeded the scope of this work and must be subject of further study. We have provided first functional evidence that CAPSL deficiency is affecting adipogenesis and differentiation in a cell model. Further experiments are needed to establish whether and how CAPSL expression is causally involved in adipose tissue overgrowth in humans.

## Materials and Methods

### Whole exome sequencing

We extracted DNA from blood and sequenced on an Illumina HiSeq. 2000 sequencing instrument. Details concerning sample preparation are provided in the Supplement. For data analysis, we used the VARBANK pipeline v.2.15 and the corresponding filter interface (unpublished, https://varbank.ccg.uni-koeln.de/). Raw reads were mapped to the human genome reference build hg19 using the Burrows Wheeler Aligner (BWA) alignment algorithm with a base quality threshold of 15 for read trimming (parameter: -q 15)^25^. The resulting binary alignment/map (BAM) files were further processed by Picard v1.64 (http://broadinstitute.github.io/picard/) to mark duplicate reads and GATK v1.6^26^ to perform local realignment around short insertions and deletions and to recalibrate the base-calling quality scores^27^.

For WES filtering, see Supplementary Information and Fig. S1.

### Patients and immunohistochemistry

We asked MSL patients who reported to the Department of Dermatology or the Department of Plastic Surgery, University Medical Center Regensburg, Germany to participate in the study by providing blood samples and/or tissue samples. For details on blood chemistry results, we refer to a recent publication by our group^28^. In short, we show that means were normal for Hba1c (5.4%), normal for HDL (61 mg/dL), slightly elevated for LDL (124.9 mg/dL, normal values < 100 mg/dL), normal for triglycerides (98 mg/dL), and a little elevated for cholesterol (210.2 mg/dL, reference value > 200 mg/dL). We stained histological sections using α-CAPSL (Sigma-Aldrich) and α-UCP1 (Abcam). We obtained biotinylated secondary antibody, streptavidin-HRP conjugate and the HRP substrate AEC from Zytomed. We stained nuclei with Hemalum solution acidic (Roth) and performed mounting using Augatex (Merck). See Details in Supplementary Information.

### Cell culture

We used 3T3-L1 pre-adipocytes to create CAPSL knockout and overexpressing cell lines. Then we differentiated cells into adipocytes using established protocols. Details are provided in the Supplementary Information (Fig. S2).

### Cell viability assay

See Supplementary Information.

### Fluorescence microscopy

Using fluorescence microscopy, we analyzed the cells after Nile Red (Sigma-Aldrich) staining or immunostaining (mounted with ProLong Diamond Antifade Mountant with Dapi (Life Technologies)). We obtained the antibodies for immunostainings from Sigma-Aldrich (α-p62, α-LC3B and α-Flag), Abcam (α-p62) and fluorescent-labeled secondary antibodies from Sigma-Aldrich and Life Technologies. To take fluorescence microscopy images, we used the Axio ImagerM2 extended with Apotom.2 (Zeiss) and analyzed with Fiji (version 2.0.0-rc-54/1.51 g). A detailed description can be found in the Supplement.

### Fluorescence activated cell sorting (FACS)

We differentiated 3T3-L1 cell lines for seven days and analyzed the adipocyte populations using flow cytometry according to a protocol published by Aldridge A *et al*. 2013. For Nile red staining, cells were harvested by treatment with 0.5% trypsin/EDTA (Invitrogen), washed two times in PBS and fixed with 10% (v/v) formalin in PBS (Sigma Aldrich). After two washes in PBS Nile Red was not prepared in methanol, DMSO was used. (Sigma Aldrich) solution (0.1 mg × mL^−1^, prepared in methanol) was added to cells at a final concentration of 0.05 μg × mL^−1^ (1:2000 dilution in PBS) and incubated at 4 °C for 20 min. Cells were washed three times with sieved and kept on ice before flow cytometry was carried out with the use of a FACS Canto I equipped with DIVA 5.0 software for analysis of data. Nile red fluorescence emission in adipogenically differentiated 3T3-L1 cells was detected on FL2 channel (bandpass filter, 585 ± 42 nm). Zombie NIR™ dye was used to discriminate live vs. dead adipocytes. Size (forward scatter, FSC) and granularity (side scatter, SSC) of cells were analyzed. Cells with increased granularity were estimated as adipocytes. 3T3-L1 populations were detected between FSC 0.9–2,5 × 10^5^. SSC between 0.4–1 × 10^5^ shows the preadipocyte population and SSC between 0.6–2.5 × 10^5^ the adipocyte population of each sample.

### Western blot

We lysed cells using a whole cell lysis buffer, digested nucleic acids and denatured the samples, then separated and blotted the protein using commercial gels and membranes. Exact conditions and materials used are described in detail in the supplement. Antibodies were purchased from Proteintech (α-CAPSL), Sigma-Aldrich (α-ß-Actin, α-HA, α-p62 and α-LC3B), Cell Signaling (α-PDI, α-AKT1, α-AMPK, α-SIRT1 and α-p70S6K (total)) and Millipore (α-p70S6K (phosphorylated)). For imaging we used the Bio-Rad ChemiDoc^TM^ MP Imaging System and normalization using total protein with Image Lab (version 5.2.1).

### Real time PCR

We extracted RNA from cells using TRI Reagent (Sigma-Aldrich). After adding 1-Bromo-3-chloropropane (Sigma-Aldrich) we obtained phase separation by centrifugation. We used the Power SYBR® Green RNA-to-CT™ 1-Step Kit (Thermo Fisher) and ran the qPCR on a StepOnePlus (Thermo Fisher). Gene expression data was generated by normalizing mRNA levels of the respective transcript to murine *Actb* mRNA levels in the case of 3T3-L1 cells, or human *ACTB* mRNA levels in the case of human adipocytes, by use of the 2^−ΔΔCT^ method. Relative fold differences in mRNA expression were calculated in relation to WT preadipocytes at the time point “d0” in the case of murine 3T3-L1 cells, and in relation to adipocytes from healthy controls at the time point “d0” in all experiments with human cells. Each data point represents triplicates. Primer sequences are listed in Table S1.

### Ethics

All experiments were conducted in accordance with the Declaration of Helsinki. The study was approved by local ethics committees (University of Regensburg ethics committee: 08/117 and 14-101-138, University of Cologne ethics committee: 13–142). Written informed consent was obtained from all participants prior to the study.

## Supplementary information

SUPPLEMENTARY INFORMATION
