# Supplementary material for: Calcyphosine-like (CAPSL) is regulated in Multiple Symmetric Lipomatosis and is involved in Adipogenesis
Source: Sci Rep. 2019 Jun 11;9:8444. doi: 10.1038/s41598-019-44382-1 (PMC6559987; doi:10.1038/s41598-019-44382-1)
Supplement: Supplementary file 1 — SUPPLEMENTARY INFORMATION [file 41598_2019_44382_MOESM1_ESM.pdf]

TITLE

Calcyphosine-like (CAPSL) is regulated in Multiple Symmetric Lipomatosis and is involved in Adipogenesis

AUTHORS

Angie Lindner[1], Felix Marbach[1], Sebastian Tschernitz[2], Christine Ortner[2], Mark Berneburg[2], Oliver Felthaus[3], Lukas Prantl[3], Min Jeong Kye[1], Gunter Rappl[4], Janine Altmüller[1,5], Holger Thiele[5], Stephan Schreml[2,#,*] and Julia Schreml[1,#]

[1]Institute of Human Genetics, University Hospital of Cologne, Germany

[2]Department of Dermatology, University Medical Center Regensburg, Germany

[3]Department of Plastic Surgery, University Medical Center Regensburg, Germany

[4]Center for Molecular Medicine Cologne (CMMC) and Department of Internal Medicine I, University of Cologne, Germany

[5]Cologne Center for Genomics (CCG), University of Cologne, Germany

Correspondence:

* E-mail: julia@schreml.de, stephan@schreml.de

# equal senior authorship

Calcyphosine-like (CAPSL) is regulated in Multiple Symmetric Lipomatosis and is involved in Adipogenesis

Angie Lindner, Felix Marbach, Sebastian Tschernitz, Christine Ortner, Mark Berneburg, Oliver Felthaus, Lukas Prantl, Min Jeong Kye, Gunter Rappl, Janine Altmüller, Holger Thiele, Stephan Schreml and Julia Schreml

SUPPLEMENTARY INFORMATION

SUPPLEMENTARY INTRODUCTION

Growing evidence has put calcium ion based signaling in the spotlight of metabolic regulation and adipose tissue differentiation. Thus, it has been observed that high extracellular calcium level inhibits 3T3-L1 adipocyte differentiation[1] and calcium binding proteins are directly involved in the mediation of differentiation signals via Peroxisome proliferator-activated receptor gamma (PPARG) and others[2]. Adipocyte differentiation is a complex multi-step process requiring a delicate interplay of major cellular processes and pathways. In early stages of differentiation, restructuring as well as adaptation of the cells for the purpose of fat storage or thermogenesis takes place[3]. For this purpose, induction of autophagy seems to be a crucial event. Differentiation of mesenchymal stem cells to mature adipocytes requires massive restructuring of cellular organelles and protein production, thereby causing stress on the intracellular metabolism. ER stress and mitochondrial dysfunction have been implicated in obesity in several mouse models[3-5]. While it has become increasingly evident that adipocyte dysfunction is linked to and can be caused by the disruption of

key regulatory processes, such as energy metabolism and growth factor signaling cascades, the exact mechanisms leading to specific adipocyte dysfunctions remain elusive.

SUPPLEMENTARY MATERIALS AND METHODS

Patients and immunohistochemistry

The Department of Dermatology, University Medical Center Regensburg, Germany and the Department of Plastic Surgery, University Medical Center Regensburg, Germany obtained blood samples for genetic testing and, where possible, subcutaneous fat tissue from MSL patients. Tissue samples were preferably taken during medically necessary interventions for MSL (e.g surgical waste from liposuction for isolation of hASCs). Similarly, we obtained control samples from healthy patients who had undergone interventions for other reasons than MSL.

Tissue samples were fixed with formalin and embedded in paraffin. Slides with paraffin sections were incubated for 30min at 72°C to melt the paraffin. Then, slides were incubated twice for 10min in Xylol (Merck), twice for 5min in 100% EtOH, 5min in 96% EtOH and 5min in 70% EtOH. After 10min incubation in 3% hydrogen peroxide, the samples that were stained with αCAPSL were washed with distilled water. Slides were then boiled 25-30min in citrate buffer pH6 (Zytomed) and cooled down for 20min at room temperature. Slides were washed in PBS (Sigma-Aldrich). All washing steps with PBS for CAPSL staining were performed for 10min und for UCP1 staining 6min. Samples were blocked with blocking solution (Zytomed) for 10min. Primary antibodies diluted in Antibody Diluent (Zytomed) were added and incubated overnight. Slides

were washed with PBS and incubated with biotinylated secondary antibody for 20min. After a wash step with PBS, streptavidin-HRP conjugate was added and samples were incubated for 20min. Slides were washed again with PBS. The HRP substrate AEC was added for around 7min and reaction was stopped with distilled water. Nuclei were stained with Hemalum solution acidic (Roth) and mounting was performed using Augatex (Merck).

Cell culture

3T3-L1 pre-adipocytes were obtained from the Applied Stem Cell Research Center University Hospital Regensburg and cultured with DMEM (Gibco) supplemented with 10% fetal calf serum (FCS) (Biochrom) and 100U/ml Penicillin/ Streptomycin (Gibco). *Capsl* knockout ($Capsl^{KO}$) cells were generated using the CRISPR/Cas9 system[1] (GeneArt® CRISPR Nuclease Vector Kit, Life Technologies) according to manufacturer's instructions. The CRISPR RNA was designed using the web server CRISPRdirect[2] and Serial Cloner (version 2.6.1). Oligonucleotides were purchased from IDT and cloned into the GeneArt® CRISPR Nuclease vector which contains CD4 reporter gene. Wild type 3T3-L1 cells were transfected with Lipofectamine (Life Technologies) and stained with α-CD4-FITC (Sigma-Aldrich). Transfected cells were sorted via FACS (Aria III, Cell Sorting Facility, CMMC) and monoclonal cell lines were examined by Sanger sequencing (CCG). The *Capsl* knockout cell line used in this study harbors the following homozygous mutation: c.21_22insT, p.D8* (NM_029341). The *Capsl* overexpressing ($Capsl^{OE}$) cell line was created using the pEGFP-N1 vector (Clontech). The construct contains c-terminal HA-tagged *Capsl* (NM_029341). $Capsl^{KO}$ cells were transfected using Lipofectamin (Life Technologies) and selected

with G418 (Sigma-Aldrich). A monoclonal cell line was obtained by FACS. The stable $Capsl^{OE}$ cell line was maintained in growth medium containing DMEM (Gibco), 10% FCS (Biochrom) and 800μg/ml G418 (Sigma-Aldrich). Human adipose derived stem cells (hASCs) were grown in αMEM (Sigma) supplemented with 0.1% GlutaMAX (Gibco), 20% FCS and 100U/ml Penicillin/ Streptomycin.

Subcutaneous fat tissue was obtained in the Department of Plastic Surgery, University Medical Center Regensburg. hASCs were isolated in the Applied Stem Cell Research Center University Hospital Regensburg. The method is described in detail elsewhere[3].

Differentiation of 3T3-L1 and hASCs to adipocytes

We obtained 3T3-L1 adipocytes by adding differentiation cocktail (Sigma-Aldrich) to two-day post-confluent cells (d0) in proliferation medium as described by Huang et al.[6]. At the fourth day (d4), we removed the medium and added proliferation medium to the cells. We harvested mature adipocytes at day 7 (d7). We induced adipogenesis in two-day post-confluent (d0) hASCs using a cocktail composed of 1μM dexamethasone, 1.7μM insulin, 0.5mM IBMX and 200μM indomethacin. We renewed the medium every second or third day for 21 days.

Cell viability assays

We treated cells as described above. We removed the medium and incubated with MTT-solution (5mg MTT/ml PBS). To dissolve formazan crystals, we used DMSO (Applichem) and measured absorbance at 550 nm using a plate reader (TECAN Safire2).

Fluorescence microscopy

Cells were fixed on coverslips for 15min at room temperature with 4% paraformaldehyde (Roth) in PBS (Gibco) and washed thrice with PBS. For lipid stainings, cells were stained with 10μg/ml Nile Red for 15min, washed thrice and mounted with ProLong Diamond Antifade Mountant with Dapi. For immunostainings to visualize CAPSL localization, fixed cells were permeabilized 15min with 0.5% Triton X-100 (Applichem) in PBS and blocked with 3% milk powder (Sigma-Aldrich) in PBS. Washing steps were performed with PBS. For LC3B and p62 stainings, fixed cells were permeabilized 5min with PBS supplemented with 0.2% Triton X-100 and blocked with 5% BSA in PBS with 0.2% Triton X-100. These cells were washed with PBS containing 0.2% Triton X-100. Coverslips for both kind of stainings, CAPSL localization and autophagy analysis, were then incubated overnight with primary antibodies. Cells were washed thrice and the respective secondary antibodies were applied in a dilution of 1:200. Following three wash steps, mounting was performed as described above.

Western Blot

Cells were lysed with 20mM Tris (pH 5.5), 150mM $NaCl_2$, 1mM EDTA, 1% NP40, 0.25% SDS, 0.1% Protease Inhibitor Cocktail (Sigma-Aldrich), 1mM $NaVo_4$ and 10mM NaF. Then, samples were centrifuged, supernatants were transferred in new tubes and nucleic acids were digested with Benzonase (Sigma-Aldrich). Protein concentration was determined via BCA assay (Thermo Fisher). Whole protein lysates were denatured with laemmli sample buffer (Bio-Rad) supplemented with 10% ß-Mercaptoethanol (Sigma-Aldrich). Proteins were loaded onto TGX Stain-Free Gels and the Trans-Blot Turbo Transfer System (Bio-Rad) was used.

Whole Exome Sequencing (WES)

DNA was randomly fragmented using sonification technology (Covaris, Inc., Woburn, MA). The fragments were end repaired and adapters were ligated. After library preparation, 6 samples were pooled and submitted to the enrichment process. We used SeqCap EZ Human Exome Library v2.0 kit following the NimbleGen SeqCap EZ Exome Library SR User's Guide. After validation (2200 TapeStation; Agilent Technologies, CA, USA), the pools were quantified using the KAPA Library Quantification kit (Peqlab, Erlangen, Germany) and the 7900HT Sequence Detection System (Applied Biosystems, Waltham, MA, USA), and subsequently sequenced on an Illumina HiSeq 2000 sequencing instrument using a paired-end 2 × 100 bp protocol and an allocation of one pool per flowcell lane.

WES filtering

For WES filtering, we used the following multi-step-strategy (**Fig. S1**). We used Varbank pre-settings and an allele frequency range of 25 - 75%. We next filtered for shared variants between all affected/the obligate carrier. This dataset was exported to Excel. Using IGV viewer interface in Varbank, we checked all 23 variants for sequencing artefacts/low quality reads. We also eliminated all variants known to the inhouse database by more than 3 hits or a frequency of more than 0.001. After this procedure, 6 variants remained. We performed functional background search using Gene Cards and OMIM (www.omim.org) and analysis of gene variants using the ExAC server

(http://exac.broadinstitute.org) and gnomAD browser (http://gnomad.broadinstitute.org)[7]. Of the 4 missense variants, *ALMS1* causes a known autosomal recessive disorder (Almstrom Syndrome) and heterozygotes are healthy. *CPNE7* shows a high number of CNV in ExAC and the missense variant in *SDC1* has 4 hits in gnomAD. The *MC1R* variant has 29 hits in gnomAD and the adjacent amino acids are also not very highly conserved. Moreover, *CPNE7*, *SDC1* and *MC1R* seemed functionally less likely. For instance, *MC1R* variants are known to be involved in normal skin pigmentation variation and specific variants are associated with a susceptibility to UV induced melanoma, however no association with adipose tissue (tumors) is described. SDC1 is a member of the syndecan proteoglycan family, is expressed in endothelial cells and may function as in trans HIV-receptors. While *SDC1* was studied in a mouse obesity model[8], in the respective study, *Sdc1* was transgenically misexpressed in the hypothalamus of mice and the phenotype was a hyperphagia induced obesity, and thereby very different from the regional tumorlike fat tissue overgrowth in MSL. *CPNE7* is predominantly expressed in the brain.

The two remaining variants were hard consequences. In *ABCB9* a frameshift was found leading to a stop at the terminal part of the protein (p.H755Lfs*2). The variant is annotated 70 times in the gnomAD browser. For *CAPSL*, a premature stop occurs at p.R9*. The same variant is annotated 5 times in the gnomAD browser. Functionally, an association with metabolism (diabetes) has

been suggested and *CAPS*, an important paralog of *CAPSL*, was repeatedly associated with tumor progression. *ABCB9* is a member of the ATP Binding Cassette Subfamily B and functions in the translocation of peptides (e.g. MHC and antigen) molecules from the cytosol into the lysosomal lumen. Mouse data available from the International mouse phenotype consortium, (http://www.mousephenotype.org/) on *Abcb9* and *Cpne7* mutants suggested no relevant pathology concerning body weight/adipose tissue. No data was available for *Capsl* and *Sdc1*. Considering all information, *CAPSL*, to us, was the most interesting candidate gene in the analyzed family.

SUPPLEMENTARY TABLE

Table S1. qPCR primer list for human and murine transcripts.

Gene	Forward Primer (5´-3´)	Reverse Primer (5´-3´)
ACTB[4]	AGAAAATCTGGCACCACACC	TAGCACAGCCTGGATAGCAA
CAPSL	ACCAGAATGGGGAATGGAGTG	GCGCTCACACCTGCATAGTA
Actb	CTGGCTCCTAGCACCATGAA	AGCTCAGTAACAGTCCGCCTA
Capsl	TGAAAGGCCAGGACAGCAAA	GATTGCCATCTCCCGGTCAT
Pparg[5]	TGTGGGGATAAAGCATCAGGC	CCGGCAGTTAAGATCACACCTAT
Cebpa[6]	CATCTGCGAGCACGAGAC	CTTGGCCTTCTCCTGCTG
Cebpb[6]	GACAAGGCCAAGATGCGCAAC	GGCAGCTGCTTGAACAAGTTC
Trim16[7]	CGGACCTCCCCAGTAGATTT	TGTGAACTCCTTCCCATTCC
Ero1-Lα	ACCCTGCCATTCTGATGAAG	GACTCATCCACGGCTCCAAG
Atf4	ACCGGCAAGGAGGATGC	TCTGGCATGGTTTCCAGGTC
Canx	TGAAGACCCAGAAGACCGGA	GGCGTCTTCATCCCAGTCAT
Erp44	CCTTGCTGCTCCTGGTAACTT	CATCTGGCTGAAACGACACC
Ire1a	CATCACTTACCCCCTGAGCG	GTAAGGTCTCCGTGTGGGTG
Pdi	GAAGAAGGAGGAGTGTCCCG	ATCAGGTGGGGCTTGATCTTG
Perk	AGGACCCTATCCTCCTGCTG	GACTCCTTCCGCTGCCTG
sXbp1	CTGAGTCCGCAGCAGGTG	CCAACTTGTCCAGAATGCCC
usXbp1	AACACGCTTGGGAATGGACA	ACATAGTCTGAGTGCTGCGG
Chop[8]	CCACCACACCTGAAAGCAGAA	GGTGCCCCCAATTTCATCT
Bcl2[8]	GGATGACTTCTCTCGTCGCTAC	TGACATCTCCCTGTTGACGCT
Grp94[9]	AAGAATGAAGGAAAAACAGGACAAAA	CAAATGGAGAAGATTCCGCC

SUPPLEMENTARY FIGURES

a

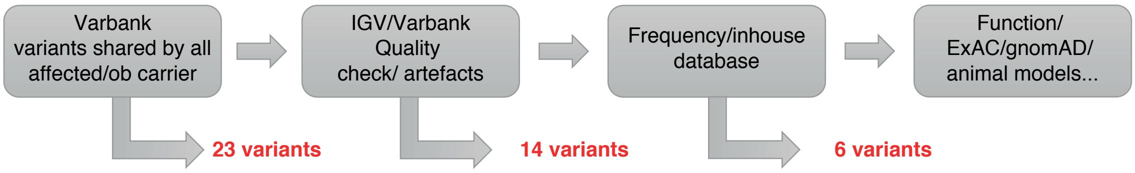

b

Gene	Variant	Protein	Consequence
ABCB9	c.2264_2273del ACAACGAGCC	p.H755Lfs*2	Frameshift
CAPSL	c.25C>T	p.R9*	Stop gained
ALMS1	c.4444A>G	p.I1482V	Missense
CPNE7	c.1579G>A	p.D527N	Missense
SDC1	c.724G>A	p.A242T	Missense
MC1R	c.363C>G	p.D121E	Missense

Figure S1. Illustration of filtering steps following Whole exome sequencing. For WES filtering a multi-step-strategy was used (**a**). Varbank standard filtering for an allele frequency range of 25-75% was performed and 23 variants were obtained that were shared between all affected/obligate carrier. A check for sequencing artefacts/low quality reads was performed using IGV viewer and Varbank reducing variants to 14. Variants known to the inhouse database by more than 3 hits or a frequency of more than 0.001 were eliminated. After this procedure 6 variants remained (**b**). These were further assessed using multiple databases (ExAC, gnomAD, OMIM, International mouse phenotype consortium, GeneCards; full detail provided in main text).

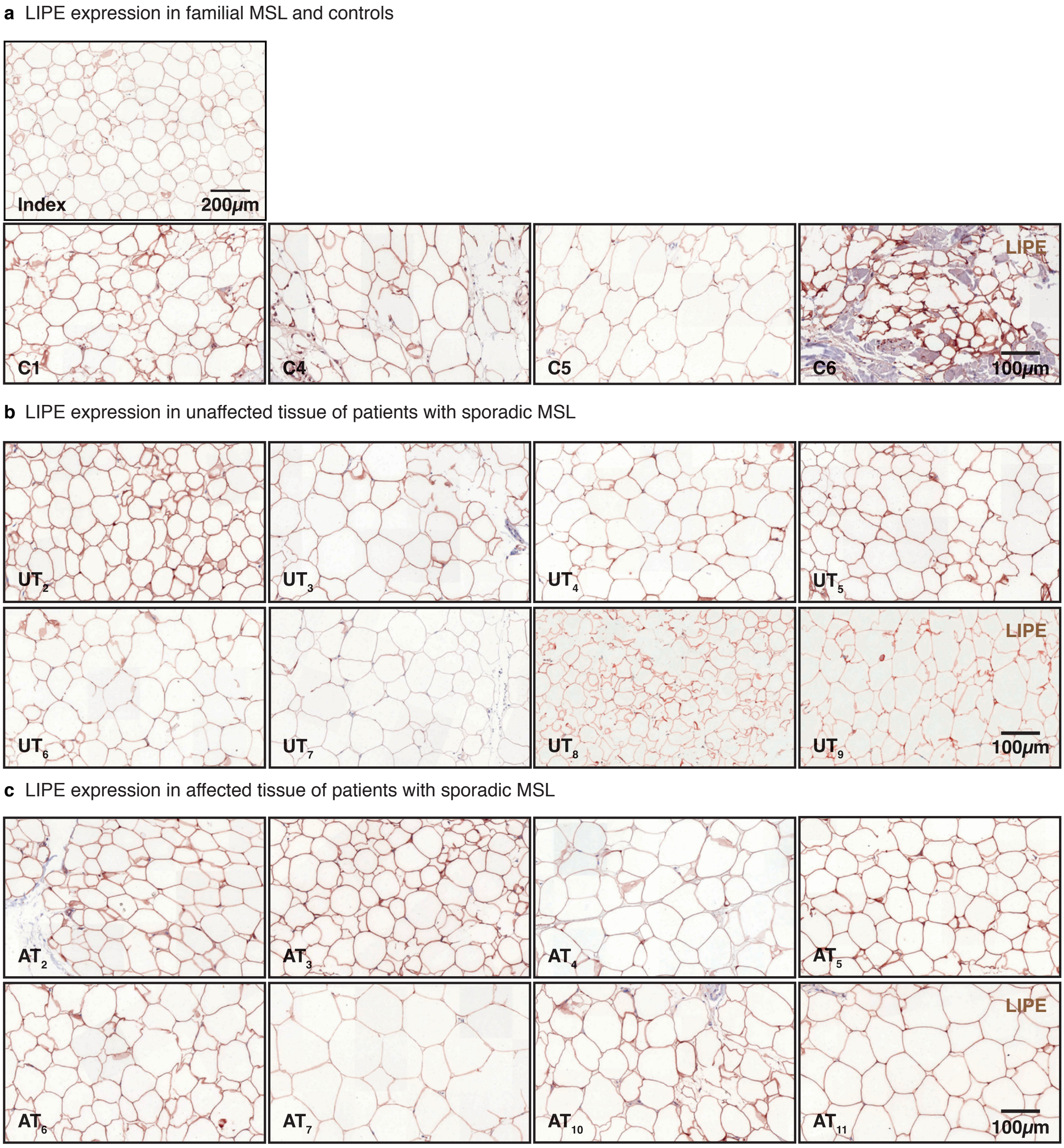

Figure S2. Immunohistochemistry of LIPE in MSL adipose tissue. LIPE expression was analyzed in control adipose tissue (**a**), index patient´s adipose tissue (**a**) as well as in unaffected (**b**) and affected (**c**) body regions of sporadic MSL. There was no difference in LIPE expression between MSL and control tissue.

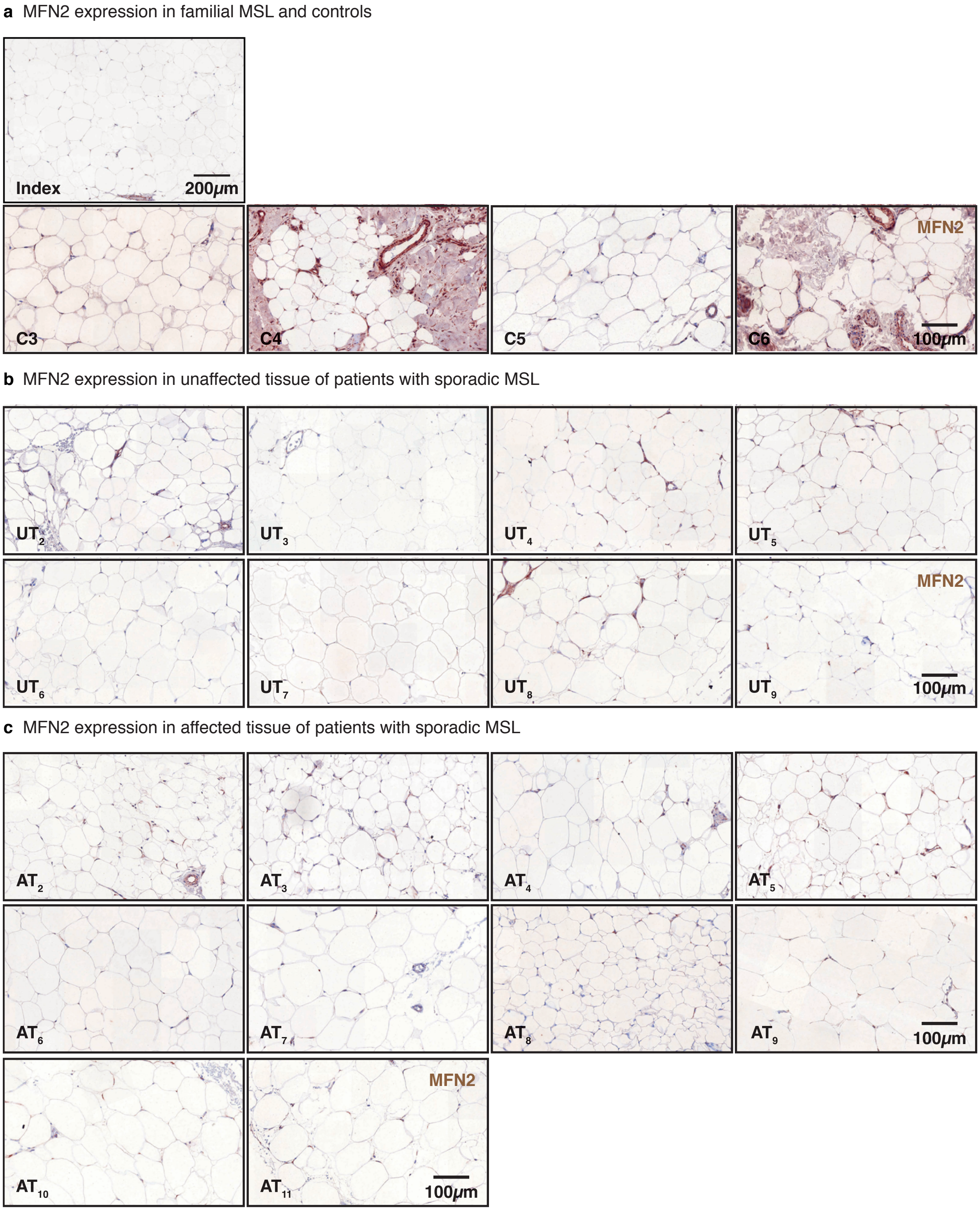

Figure S3. Immunohistochemistry of MFN2 in MSL adipose tissue. MFN2 expression was analyzed in control adipose tissue (**a**), index patient´s adipose tissue (**a**) as well as in unaffected (**b**) and affected (**c**) body regions of sporadic MSL. MFN2 was not or scarcely expressed in controls and MSL patients with two controls

showing overstaining or slightly higher expression than other samples.

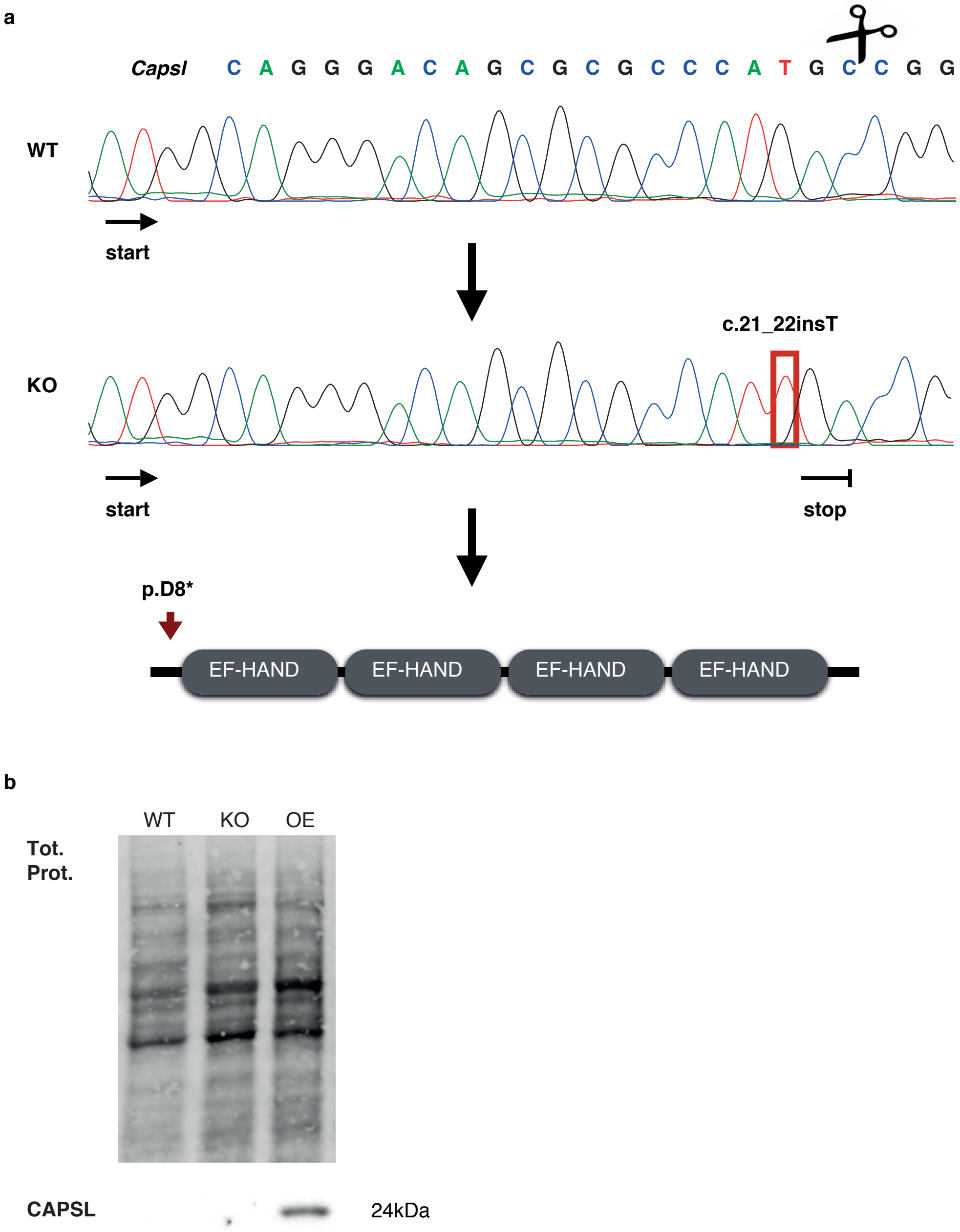

Figure S4. Generation of 3T3-L1 *Capsl* cell line models. a. A *Capsl* knockout cell line (KO) was created using wild type 3T3-L1 cells (WT) and the CRISPR/CAS9 system, causing an early stop codon at position p.D8* (NM_029341). **b.** Western Blot analysis of the *Capsl* overexpression (OE) cell line, generated by stable transfection. Endogenous *Capsl* could not be detected in WT cells.

a

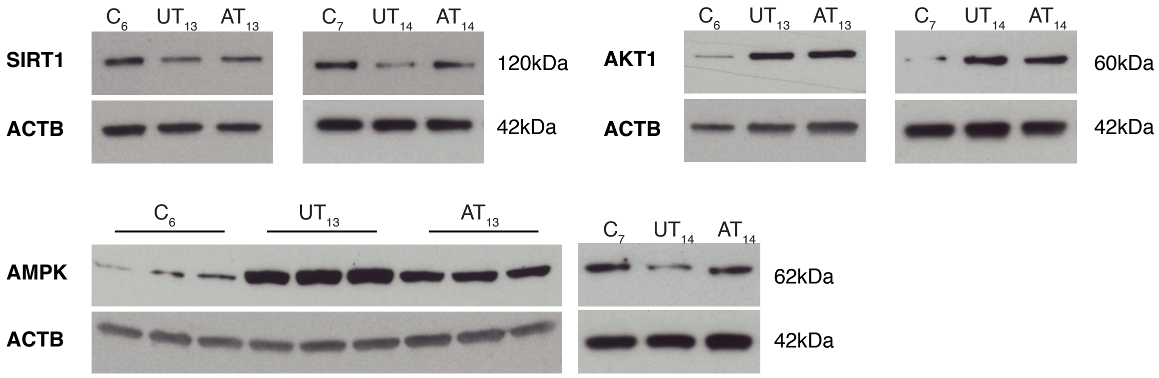

b

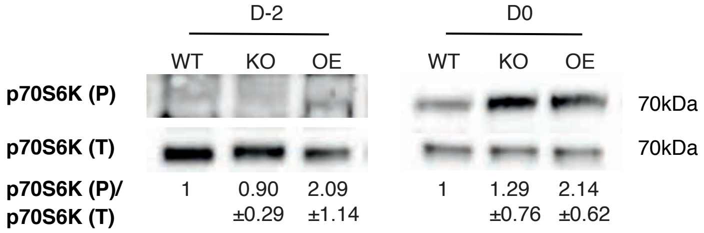

Figure S5 a. Analysis of proteins involved in adipogenesis and metabolism in *in vitro* differentiated MSL hASCs. Adipocytes from control patients (n=2) and MSL affected patients (n=2) extracted from unaffected (UT) and affected (AT) tissue were investigated. Results shown for 3 independent experiments for each patient culture for SIRT1 (P_{13}, P_{14}: n=3), AKT1 (P_{13}, P_{14}: n=4) and AMPK (P_{13}: n=2, P_{14}: n=3). **b. mTOR activity in *Capsl* deficient (KO) and overexpressing (OE) cells.** p70S6K phosphorylated and total protein levels were examined in proliferating cells (d-2) and at the beginning of differentiation (d0) using western blots (n=3, mean ± SD)**.** Basal levels could barely be detected in all cell lines.

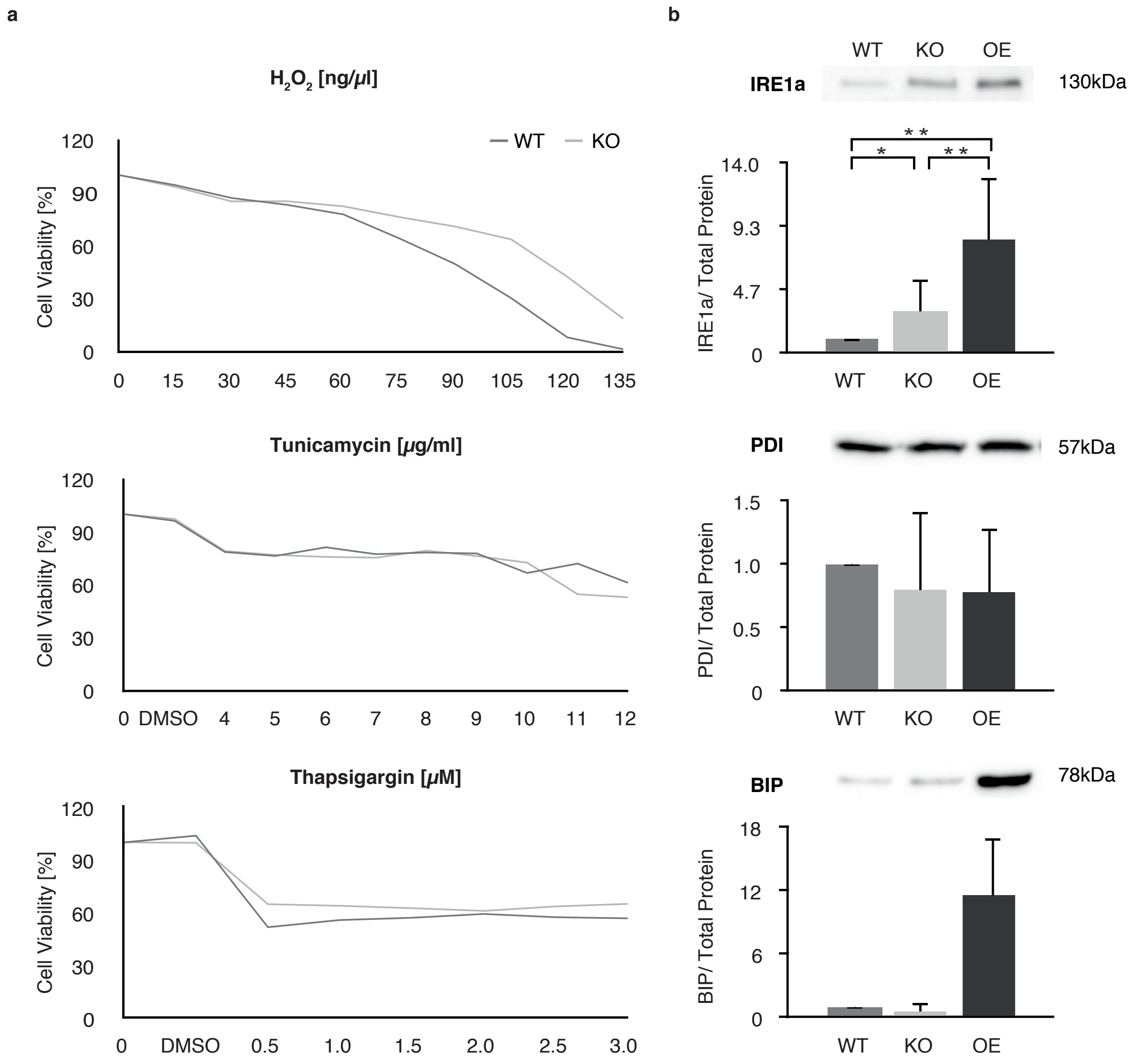

Figure S6. Oxidative and ER stress in 3T3-L1 *Capsl* deficient (KO) and overexpressing (OE) cell lines. a. Cell viability was evaluated using MTT assays after treatment with H_2O_2 (1h in H_2O_2 and 18h in proliferation medium, n=3), Tunicamycin (5h, n=3) and Thapsigargin (20h, n=4) with the indicated concentrations. **b.** Immunoblot analysis of the ER stress markers (n=3) IRE1a, BIP and PDI in wild type (WT), KO and OE cells were performed. Statistical significance was evaluated using student´s t-test (*$p < 0.05$, **$p < 0.01$, mean ± SD).

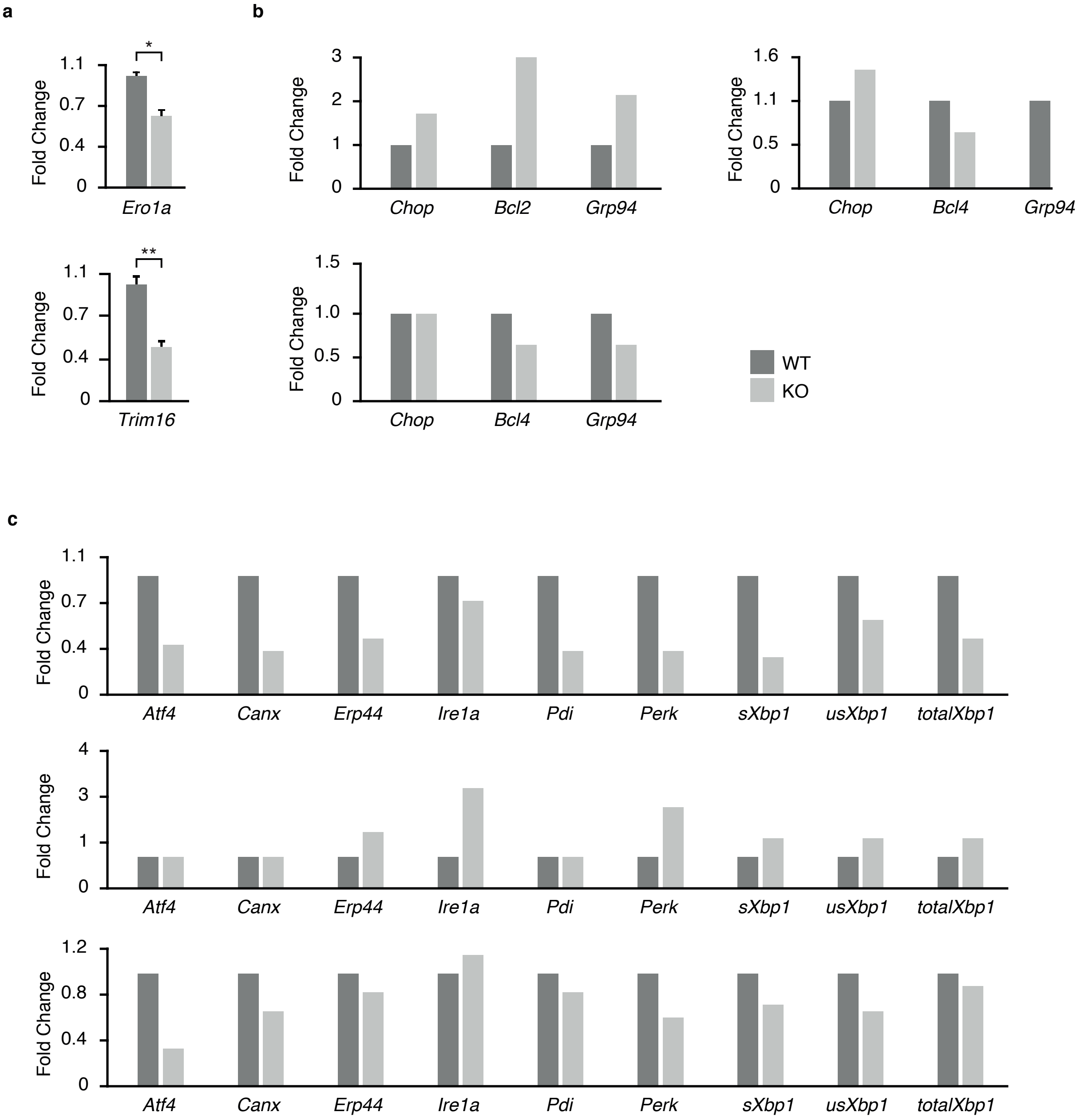

Figure S7. Gene expression levels of ER stress markers in *Capsl* deficient 3T3-L1 cells (KO). qPCR of *Capsl* deficient and WT cells at baseline conditions showed a significant decrease in Endoplasmic reticulum oxidoreductase 1 alpha (*Ero1a*) and Tripartite motif containing 16 (*Trim16*) levels (**a**), but not in the expression of additional markers tested, including Protein disulfide isomerase associated 3 (*Pdi*), DNA-damage inducible transcript 3 (*Chop*) and Activating transcription factor 4 (*Atf4*) (**b-c**).

a

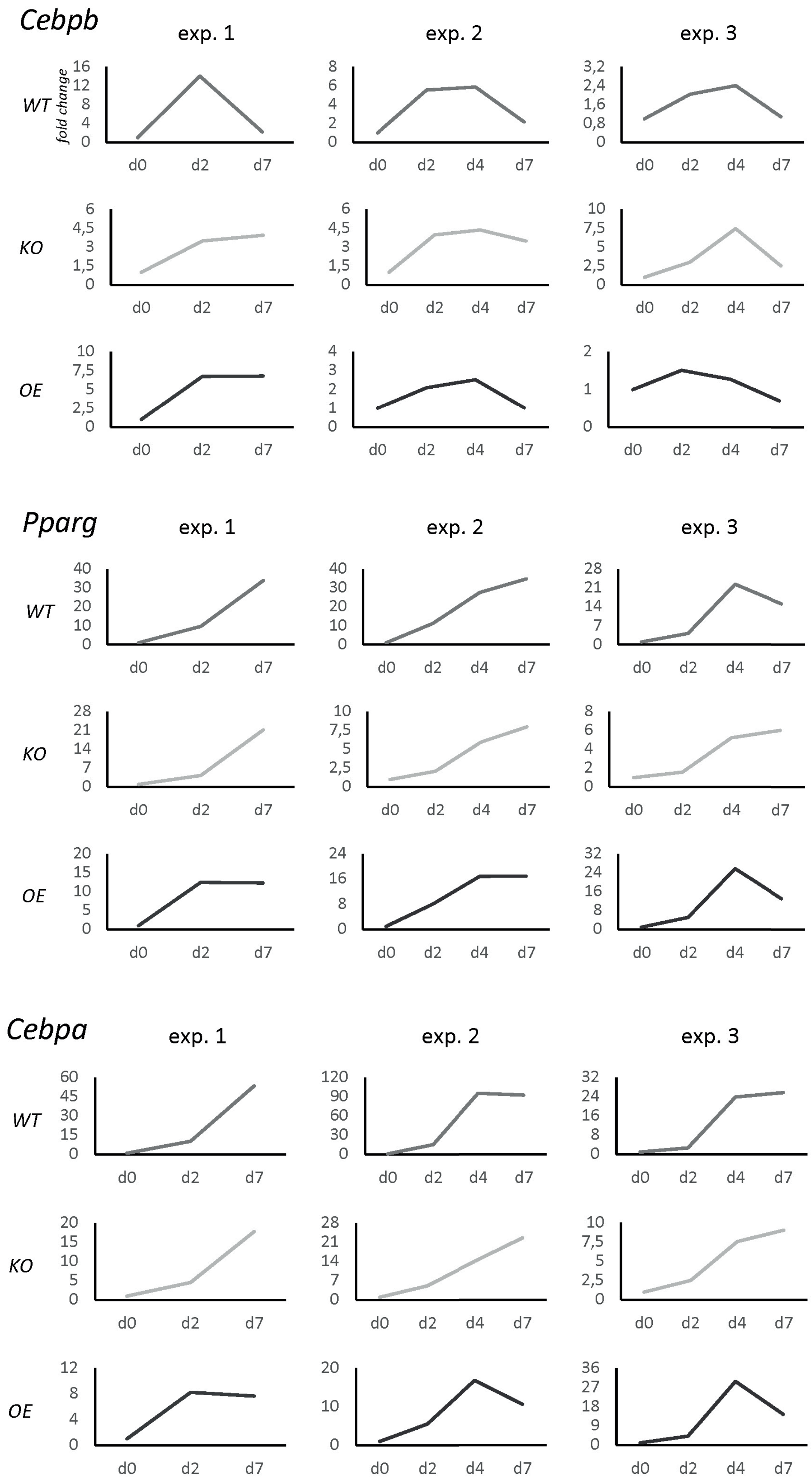

Cebpb
exp. 1
exp. 2
exp. 3
WT
KO
OE
fold change
d0
d2
d4
d7
Pparg
exp. 1
exp. 2
exp. 3
WT
KO
OE
Cebpa
exp. 1
exp. 2
exp. 3
WT
KO
OE

b

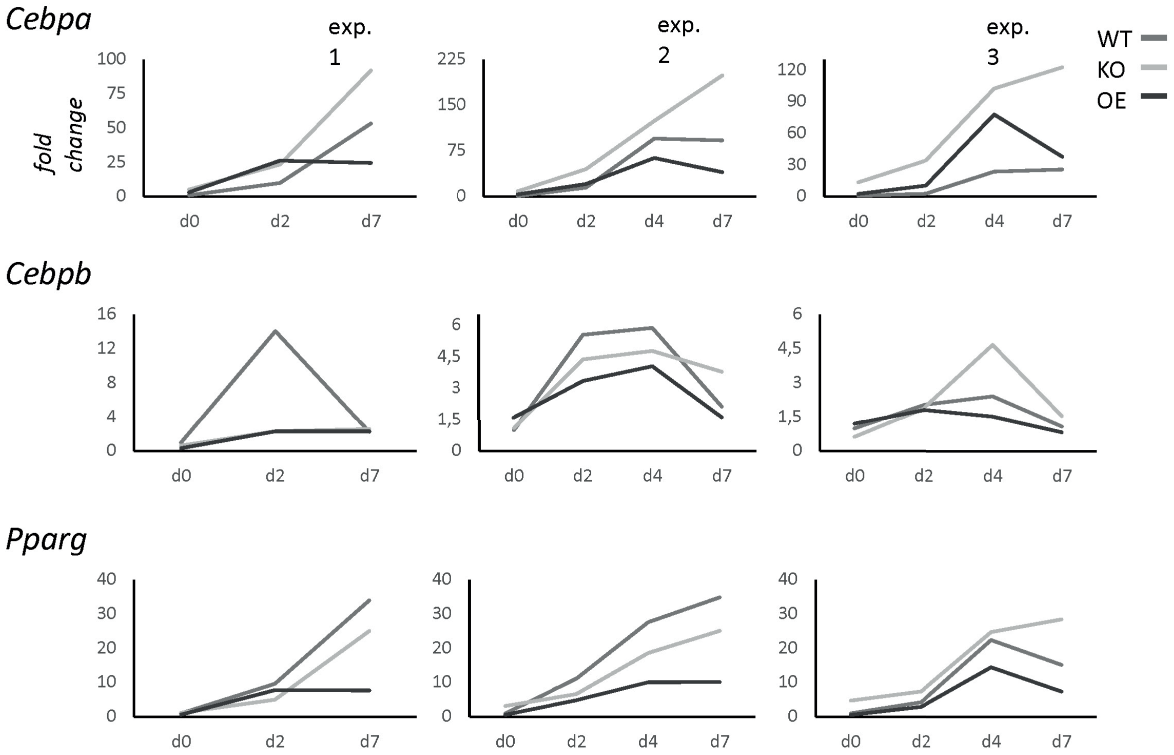

Figure S8. Gene expression levels of *Cebpa, Cebpb*, *Pparg* and *Capsl* during adipocyte differentiation of wild-type (WT), *Capsl* deficient (KO) and overexpressing (OE) 3T3-L1 cells

a. Expression of the adipocyte differentiation markers *Cebpa, Cebpb* and *Pparg* in 3T3-L1 cells (WT, KO and OE) over the course of 3 separate experiments. Fold change values are calculated in relation to the respective cell line (d0).

b. Expression of adipocyte differentiation markers in WT, KO and OE cells over the same 3 experiments, fold change values of each cell type are now calculated in relation to WT cells (d0).

UNCROPPED WESTERN BLOTS

Figure 4.

f

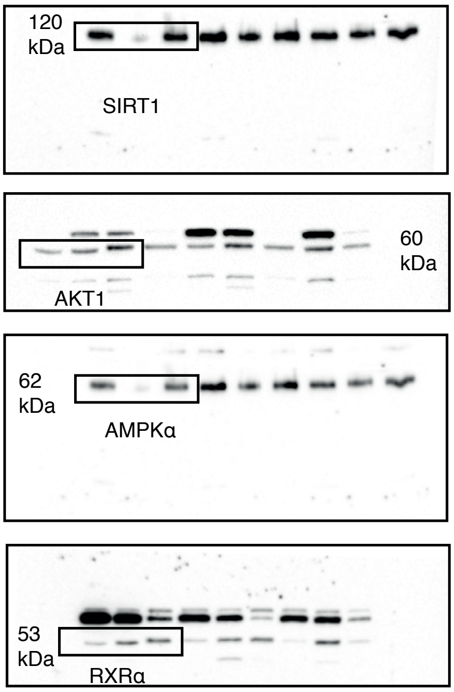

g

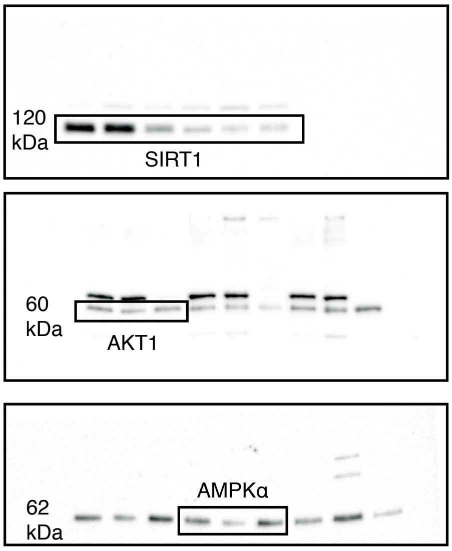

Figure 5.

a

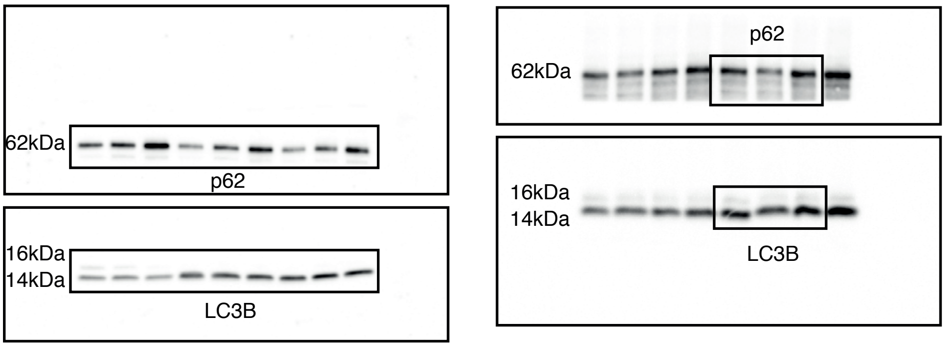

Figure 6.

a

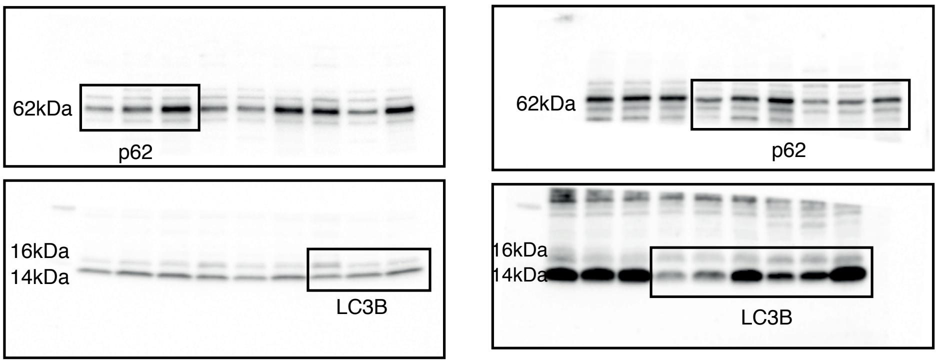

Figure S4.

b

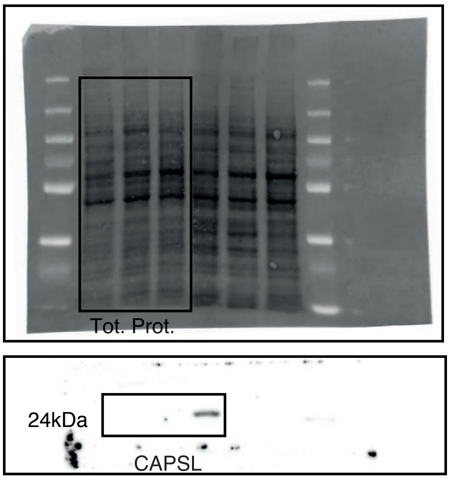

Figure S5.

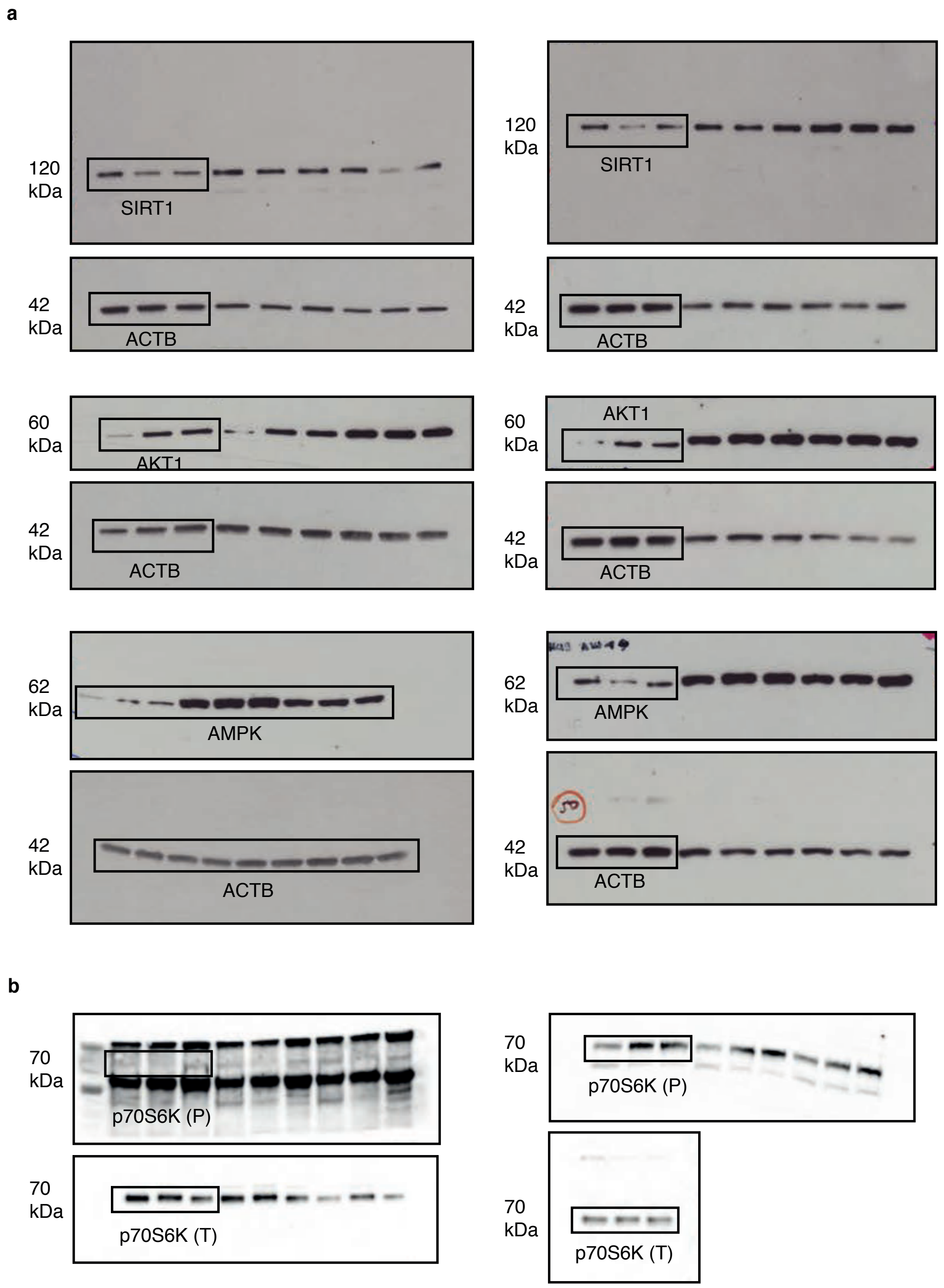
a
120 kDa
SIRT1
42 kDa
ACTB
60 kDa
AKT1
62 kDa
AMPK
b
70 kDa
p70S6K (P)
p70S6K (T)

Figure S6.

b

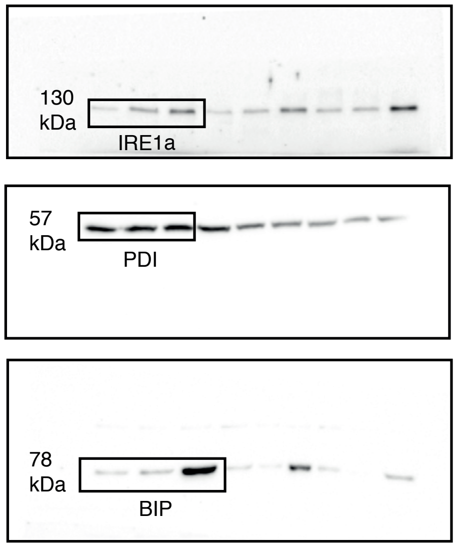

SUPPLEMENTARY REF ERENCES

1 Jensen, B., Farach-Carson, M. C., Kenaley, E. & Akanbi, K. A. High extracellular calcium attenuates adipogenesis in 3T3-L1 preadipocytes. *Exp Cell Res* **301**, 280-292, doi:10.1016/j.yexcr.2004.08.030 (2004).

2 Lin, F., Ribar, T. J. & Means, A. R. The Ca2+/calmodulin-dependent protein kinase kinase, CaMKK2, inhibits preadipocyte differentiation. *Endocrinology* **152**, 3668-3679, doi:10.1210/en.2011-1107 (2011).

3 Rabiee, A. *et al.* Nuclear phosphoproteome analysis of 3T3-L1 preadipocyte differentiation reveals system-wide phosphorylation of transcriptional regulators. *Proteomics* **17**, doi:10.1002/pmic.201600248 (2017).

4 Yang, L. *et al.* METABOLISM. S-Nitrosylation links obesity-associated inflammation to endoplasmic reticulum dysfunction. *Science* **349**, 500-506, doi:10.1126/science.aaa0079 (2015).

5 Hsu, H. C. *et al.* Time-dependent cellular response in the liver and heart in a dietary-induced obese mouse model: the potential role of ER stress and autophagy. *Eur J Nutr* **55**, 2031-2043, doi:10.1007/s00394-015-1017-8 (2016).

6 Huang, C. *et al.* Berberine inhibits 3T3-L1 adipocyte differentiation through the PPARgamma pathway. *Biochem Biophys Res Commun* **348**, 571-578, doi:10.1016/j.bbrc.2006.07.095 (2006).

7 Lek, M. *et al.* Analysis of protein-coding genetic variation in 60,706 humans. *Nature* **536**, 285-291, doi:10.1038/nature19057 (2016).

8 Reizes, O. *et al.* Transgenic expression of syndecan-1 uncovers a physiological control of feeding behavior by syndecan-3. *Cell* **106**, 105-116 (2001).
